# Crystal Structure Prediction and 0 K Stability Assessment of Hypothetical Re_3_Zr Polymorphs: Vibrational, Elastic, and Electronic Properties

**DOI:** 10.3390/ma19183844

**Published:** 2026-09-09

**Authors:** Mardan Mamatyusup, Qun Wei, Zhengzhe Lin, Jing Luo, Meiguang Zhang

**Affiliations:** 1 School of Physics, Xidian University, Xi’an 710071, China; mardan@stu.xidian.edu.cn (M.M.); zzlin@xidian.edu.cn (Z.L.); luoj0225@163.com (J.L.); 2 College of Physics and Optoelectronic Technology, Baoji University of Arts and Sciences, Baoji 721016, China

**Keywords:** Re_3_Zr, crystal structure prediction, first-principles calculations, elastic anisotropy, Debye temperature, electronic structure

## Abstract

**Highlights:**

- CALYPSO identifies five alternative hypothetical Re_3_Zr polymorphs at ambient pressure.
- *Cmcm* is the lowest-energy Re_3_Zr polymorph and lies 0.042 eV atom^−1^ above the selected-phase partial hull.
- All six polymorphs are dynamically and mechanically stable local minima at 0 K.
- *P*4/*mmm* has the highest elastic Debye temperature, whereas *Fm*-3*m* is the least anisotropic.
- All six polymorphs are metallic, with Fermi-level states dominated by Re-5d orbitals.

**Abstract:**

Re_3_Zr has recently been proposed in the cubic *Pm*-3*n* structure. Its alternative atomic arrangements and thermodynamic position within the Re–Zr system have not been systematically resolved. In this work, CALYPSO particle-swarm structure searches combined with density functional theory identify five Re_3_Zr polymorphs with *Cmcm*, *Fmmm*, *Immm*, *P*4/*mmm*, and *Fm*-3*m* symmetries and compare them consistently with the reported *Pm*-3*n* phase. Formation enthalpies, a selected-phase partial convex hull, phonon dispersions, elastic properties, sound velocities, elastic Debye temperatures, electronic structures, and bonding characteristics are evaluated. All five CALYPSO structures lie below *Pm*-3*n* in fixed-composition energy, with *Cmcm* being the lowest. The *Cmcm* phase has a formation enthalpy of −0.257 eV atom^−1^ and lies approximately 0.042 eV atom^−1^ above the selected-phase partial hull, identifying it as a low-lying off-hull metastable candidate at 0 K. AIMD simulations at 300 and 1000 K show no obvious structural reconstruction of the *Cmcm* framework over the simulated time scales. All six structures exhibit no imaginary phonon frequencies and satisfy the corresponding mechanical-stability criteria. Their elastic responses are strongly structure dependent: *P*4/*mmm* has the largest Chen–Tian hardness estimates and elastic Debye temperature, whereas *Fm*-3*m* shows the weakest elastic anisotropy. All six polymorphs are metallic, with Fermi-level states dominated by Re-5d orbitals and predominantly delocalized bonding. These results provide a thermodynamically constrained 0 K reference for future synthesis, phase identification, and finite-temperature or kinetic investigations of hypothetical Re_3_Zr polymorphs.

## 1. Introduction

Advanced aero-engine and gas-turbine hot-section components require alloys that retain load-bearing capacity and microstructural stability at elevated temperatures. Rhenium and zirconium can influence these requirements through distinct mechanisms. Re additions enhance the creep strength of advanced Ni-based superalloys [1], whereas controlled Zr additions can strengthen grain boundaries and prolong creep life [2]. Re has also been shown to strengthen NbTiZr at elevated temperatures and improve the ductility of TaTiZr over a broad temperature range [3]. These observations make reliable knowledge of Re-Zr phase equilibria and crystal structures relevant to understanding structure-property relationships in refractory and high-temperature alloy systems. Experimental descriptions of the binary system have evolved from the preparation of hexagonal Re_2_Zr and an early phase diagram [4,5] to the identification of rhombohedral Re_25_Zr_21_ [6]. Subsequent CALPHAD and experimental reassessments considered or confirmed Re_24_Zr_5_, Re_2_Zr, Re_25_Zr_21_, and ReZr_2_ and refined the accepted phase relations [7,8,9]. This history shows that phase identification in the Re-Zr system depends on the combined resolution of crystallographic and thermodynamic evidence, while physical-property data remain uneven among the known compounds.

First-principles calculations have complemented these experimental studies. Levy et al. recovered Re_24_Zr_5_, Re_2_Zr, and Re_25_Zr_21_ as stable entries in a high-throughput survey of ordered Re binary alloys [10], and Kong et al. examined the elastic anisotropy, hardness, and thermal descriptors of Re_2_Zr [11]. More recently, Pan et al. compared five Re-Zr compositions and proposed cubic ReZr and Re_3_Zr models [12]. Their A15-type *Pm*-3*n* Re_3_Zr structure exhibited no imaginary phonon frequencies and satisfied the mechanical-stability criteria, although their convex-hull analysis placed Re_3_Zr above the 0 K hull. Moreover, the structure was obtained by optimizing a predefined AB_3_ prototype rather than through a global structure search. Its phonon and elastic stability therefore establish a local minimum but do not determine whether it is the lowest-energy arrangement at the Re_3_Zr composition or thermodynamically stable against decomposition into other Re-Zr phases.

Although several equilibrium compounds have been established in the Re-Zr system, Re_3_Zr remains a theoretical composition whose polymorphic landscape has not been systematically explored. Resolving this structure-prediction problem complements the continued characterization of the established phases. Motivated by the *Pm*-3*n* model proposed by Pan et al. [12], we treat Re_3_Zr as a hypothetical fixed-composition system and perform a prototype-independent global search to identify competing low-energy atomic arrangements. The predicted structures and their crystallographic, vibrational, mechanical, and electronic signatures provide a coherent theoretical reference for future experimental searches, phase identification, and subsequent finite-temperature and kinetic assessment.

In this work, CALYPSO particle-swarm optimization [13,14] is combined with density-functional calculations to explore Re_3_Zr at 0 K and ambient pressure. Five candidate structures with *Cmcm*, *Fmmm*, *Immm*, *P*4/*mmm*, and Fm-3m symmetries are identified and compared consistently with the reported *Pm*-3*n* phase. Formation enthalpies and a selected-phase partial convex hull are used to assess the thermodynamic competitiveness of the predicted structures relative to the selected competing Re-Zr phases and to distinguish this assessment from fixed-composition energy ordering. Phonon and elastic calculations are used to assess dynamical and mechanical stability. The elastic moduli, empirical hardness, anisotropy, sound velocities, elastic Debye temperatures, electronic structures, and bonding characteristics are then evaluated as comparative descriptors. Integrated first-principles approaches of this type are widely used to examine structural stability and associated elastic, vibrational, and electronic behavior across diverse materials [15,16,17,18]. Together, these calculations provide an internally consistent 0 K assessment of the hypothetical Re_3_Zr polymorphic landscape.

## 2. Computational Methods

Crystal-structure searches for fixed-composition Re_3_Zr were performed at 0 K and ambient pressure using CALYPSO (v4.0) [13,14], which employs particle swarm optimization combined with symmetry-constrained random structure generation. Cells containing 1, 2, and 4 formula units were used to initialize the searches. Three independent searches were conducted, with an average of 15 generations per search, 50 candidates per generation, and a PSO ratio of 0.6. All candidates were relaxed using a four-stage coarse-to-fine procedure. The lowest-energy structure was reproduced at least twice in each independent search. The previously proposed *Pm*-3*n* phase [12] was included for comparison and fully reoptimized using the same settings.

All first-principles calculations were performed within density functional theory using the Vienna Ab initio Simulation Package (VASP 6.1.0) [19]. The exchange-correlation interaction was described by the Perdew-Burke-Ernzerhof generalized gradient approximation [20], and the electron-ion interaction was treated using the projector-augmented-wave method [21]. For Zr, the PAW_PBE Zr_sv potential (4 January 2005) was used, with the 4s^2^, 4p^6^, 4d^3^, and 5s^1^ states treated as valence states (12 valence electrons). For Re, the PAW_PBE Re potential (17 January 2003) was used, with the 5d^6^ and 6s^1^ states treated as valence states (7 valence electrons). A plane-wave cutoff energy of 600 eV was used. Γ-centered *k*-point meshes were generated with VASPKIT 1.5.1 [22] at a reciprocal-space resolution of 0.01 Å^−1^. First-order Methfessel–Paxton smearing [23] with a width of 0.2 eV was used. The lattice vectors and atomic positions were fully relaxed until the total-energy change was below 1.0 × 10^−8^ eV and the residual forces were below 0.01 eV Å^−1^. Ab initio molecular dynamics (AIMD) simulations were further performed for the lowest-energy *Cmcm* phase in the canonical ensemble using the Langevin thermostat. Simulations were carried out at 300 and 1000 K with a time step of 2 fs, using a fixed simulation cell and a 2 × 2 × 2 k-point mesh. The trajectories extended to 10.0 ps at 300 K and approximately 9.6 ps at 1000 K.

The energy–volume relations of the six Re_3_Zr phases were obtained by uniformly scaling the linear lattice dimensions from −5% to +5%, corresponding to an approximate volume range of −14.3% to +15.8%, and fitting the calculated energies to the third-order Birch–Murnaghan equation of state [24]. Phonon dispersions for all six Re_3_Zr phases were calculated using the finite-displacement method implemented in PHONOPY [25]. Because a common replication factor produces different total numbers of atoms for structures with different *Z* values, where *Z* denotes the number of Re_3_Zr formula units in the unit cell, phase-dependent supercells were adopted, with every finite-displacement supercell containing at least 96 atoms. The second-order elastic constants were determined by the stress–strain method. Polycrystalline elastic properties were obtained using the Voigt-Reuss-Hill approximation [26,27,28], while the universal anisotropy index, directional elastic moduli, and Vickers hardness were evaluated using the established anisotropy relations [29] and the Chen [30] and Tian [31] models. Sound velocities and Debye temperatures were derived from the elastic moduli and densities [32]. Band structures were evaluated along high-symmetry paths, while total and orbital-projected densities of states used Γ-centered meshes at the same resolution; electron localization functions [33] were obtained with VASP [19]. Chemical bonding in *Cmcm* and Re_2_Zr was analyzed using the COHP method implemented in LOBSTER 5.1.1 [34,35]. The pbeVaspFit2015 basis set was used with Re 5d/6s and Zr 4s/4p/4d/5s orbitals. COHP calculations included interatomic distances up to 3.3 Å with a Gaussian smearing of 0.05 eV. The absolute charge spilling was 3.63% for *Cmcm* and 3.28% for Re_2_Zr. Bonding strengths are reported as −ICOHP, with larger positive values indicating stronger net bonding interactions.

## 3. Results and Discussion

### 3.1. Crystal Structures of Re_3_Zr Phases

**Figure 1:**
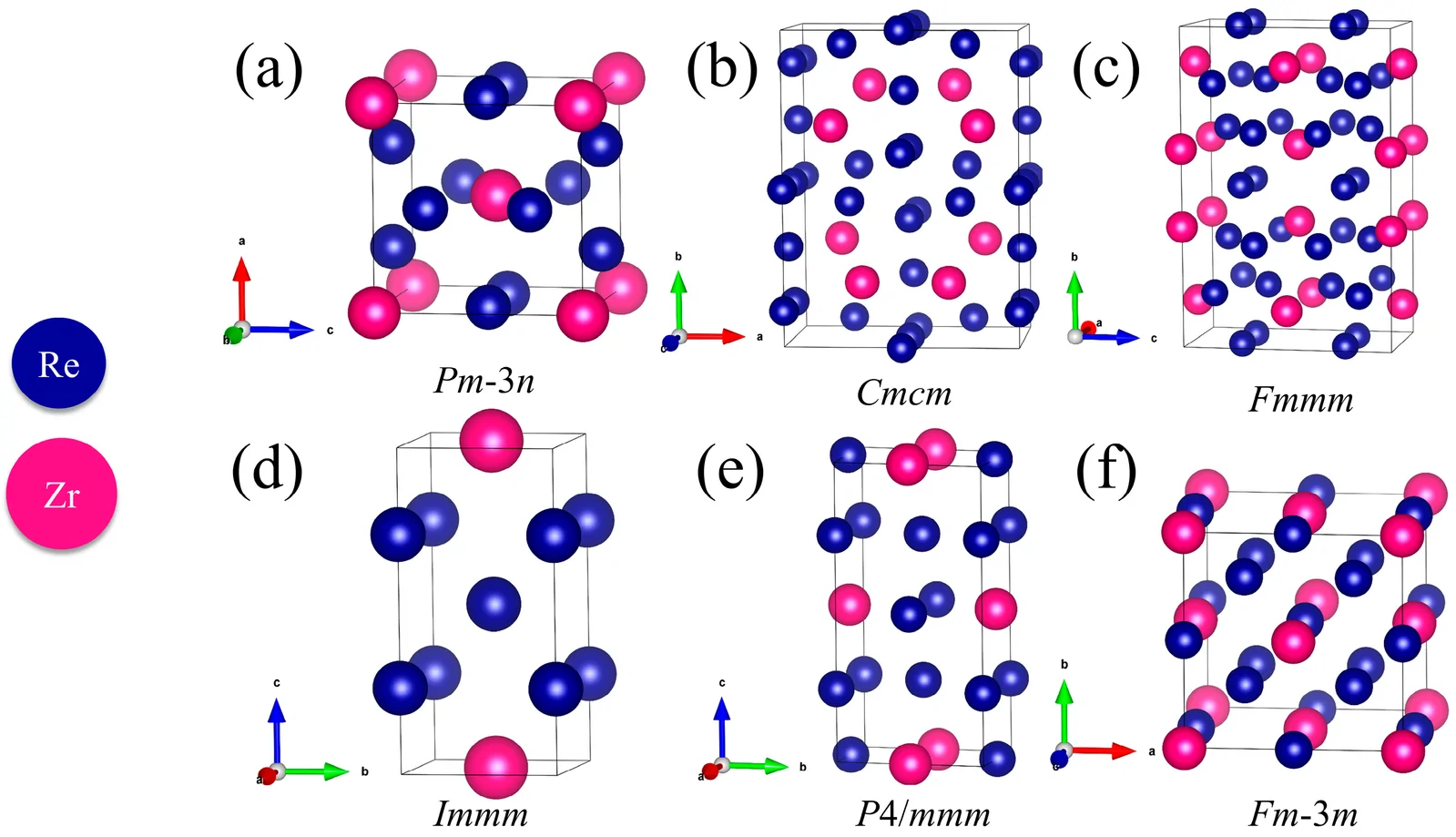
Optimized crystal structures of the six Re_3_Zr polymorphs considered in this work: (**a**) *Pm*-3*n*, (**b**) *Cmcm*, (**c**) *Fmmm*, (**d**) *Immm*, (**e**) *P*4/*mmm*, and (**f**) *Fm*-3*m*. Dark-blue and magenta spheres denote Re and Zr atoms, respectively.

For Re–Zr intermetallics, crystal structure governs atomic packing and bonding and therefore underpins phase stability and mechanical response. A recent first-principles study considered Re_3_Zr in the cubic *Pm*-3*n* structure [12]. In that work, however, the A15 (Cr_3_Si-type) structure was treated as a predefined candidate and subsequently optimized rather than identified through an unbiased global structure search. Other competitive Re_3_Zr configurations may therefore have been overlooked. To explore the structural landscape more comprehensively, a global search was performed at ambient pressure using the particle swarm optimization algorithm implemented in CALYPSO. Five candidate phases with *Cmcm*, *Fmmm*, *Immm*, *P*4/*mmm*, and *Fm*-3*m* symmetries were identified and evaluated alongside the reported *Pm*-3*n* phase. Their optimized structures are shown in Figure 1, the principal crystallographic parameters are listed in Table 1, and the nonequivalent atomic sites and local coordination environments are summarized in Table 2. The fully optimized lattice vectors and fractional atomic coordinates of all six Re_3_Zr polymorphs are provided in the Supplementary Materials in VASP POSCAR format (Tables S1–S6). These structural results provide the basis for the subsequent stability and mechanical-property analyses.

To clarify how crystal symmetry changes the packing of Re_3_Zr at fixed composition, the global crystallographic features of the six phases are first compared. The structures span three crystal systems: orthorhombic *Cmcm*, *Fmmm*, and *Immm*; tetragonal *P*4/*mmm*; and cubic *Pm*-3*n* and *Fm*-3*m*. Their conventional cells contain between one and eight formula units. The *Cmcm* and *Fmmm* phases have the largest cells, each containing eight formula units, whereas *P*4/*mmm* contains only one. The volume per formula unit, which enables a direct packing comparison among cells of different sizes, ranges from 65.04 Å^3^ for *Fm*-3*m* to 69.53 Å^3^ for *Fmmm*. Correspondingly, *Fm*-3*m* has the highest density of 16.59 g cm^−3^, while *Fmmm* has the lowest value of 15.52 g cm^−3^. The remaining four phases lie within a narrower density range of 15.98–16.52 g cm^−3^. Thus, even at the same stoichiometry, the alternative symmetries produce appreciable differences in packing efficiency.

Because global lattice parameters alone do not reveal the bonding topology that controls stability and deformation, the local coordination environments were examined further (Table 2). Here, Zr*_x_*Re*_y_* denotes a coordination environment containing *x* Zr and *y* Re nearest neighbors, and CN is the total coordination number. The *Cmcm* phase contains five nonequivalent sites with CN values of 12–16 and the broadest bond-length interval of 2.60–3.08 Å, indicating a heterogeneous local environment. The *Fmmm* phase also shows mixed coordination, with CN = 8–12 and bond lengths of 2.57–3.04 Å. By contrast, all nonequivalent sites in *Immm* and *P*4/*mmm* are eightfold coordinated, and their narrow bond-length ranges of 2.72–2.82 and 2.72–2.83 Å, respectively, indicate more uniform first-neighbor environments. In the A15-type *Pm*-3*n* phase, Zr at the 2a site is surrounded by 12 Re atoms, whereas Re at the 6d site has four Zr and two Re nearest neighbors. The *Fm*-3*m* phase exhibits eightfold coordination at all nonequivalent sites and a single nearest-neighbor distance of 2.76 Å, representing the most uniform local geometry among the six structures. The contrast between these heterogeneous and uniform coordination motifs provides an atomistic basis for interpreting the energetic, vibrational, and elastic responses discussed below.

Before ranking the competing phases, the reported *Pm*-3*n* structure provides a useful internal benchmark for the present calculations. Its optimized lattice constant of 5.113 Å differs from the previous theoretical value of 5.116 Å by only 0.06%, and the corresponding densities are 16.15 and 16.12 g cm^−3^, respectively [12]. This close agreement validates the present structural-relaxation settings for the reference model. It does not, however, establish *Pm*-3*n* as the preferred phase. The pronounced differences in cell size, packing density, and local coordination among the six candidates therefore motivate the energetic, dynamical, and mechanical stability assessments presented in the following subsection.

### 3.2. Stability and Mechanical Properties

**Figure 2:**
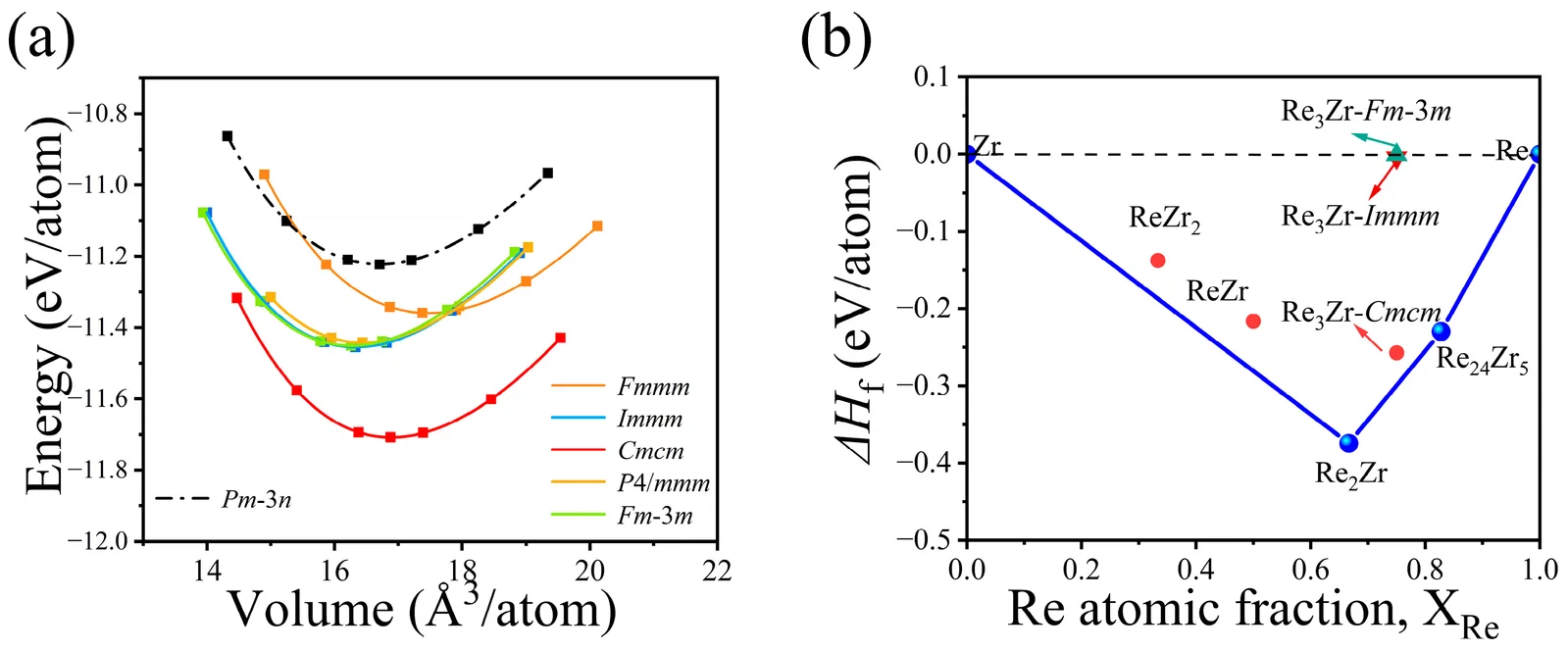
(**a**) Calculated energy–volume data and corresponding third-order Birch–Murnaghan equation-of-state fits for the six Re_3_Zr polymorphs. The dashed curve denotes the previously reported *Pm*-3*n* structure, whereas the solid curves represent the five structures identified by CALYPSO. (**b**) Calculated formation enthalpies and selected-phase partial convex hull of the Re–Zr system at 0 K and 0 GPa. The blue line and blue symbols denote the lower envelope and its vertices within the selected phase set, whereas the remaining symbols denote structures above the partial hull.

Static energy ordering among polymorphs at a fixed composition and thermodynamic stability against decomposition into competing phases address different questions. We therefore first compare the energy–volume relations of the six Re_3_Zr polymorphs and then place them in the compositional context of the Re–Zr system using consistently calculated formation enthalpies and a selected-phase partial convex hull. The energy–volume data were fitted using the third-order Birch–Murnaghan equation of state (EOS) [24]:

(1) $$E\left(V\right)=E_{0}+\frac{9V_{0}B_{0}}{16}\left\{{\left[{\left(\frac{V_{0}}{V}\right)}^{\frac{2}{3}}-1\right]}^{3}B_{0}^{\prime }+{\left[{\left(\frac{V_{0}}{V}\right)}^{\frac{2}{3}}-1\right]}^{2}\left[6-4{\left(\frac{V_{0}}{V}\right)}^{\frac{2}{3}}\right]\right\}$$

where *E*_0_, *V*_0_, *B*_0_, and *B*_0_′ are the equilibrium energy, equilibrium volume, bulk modulus, and pressure derivative of the bulk modulus, respectively. The resulting energy–volume relations describe the fixed-composition energetic ordering of the six Re_3_Zr polymorphs and their response to isotropic volume changes. As shown in Figure 2a, the calculated energy–volume points are smoothly reproduced by the EOS fits. The *Cmcm* phase has the lowest equilibrium energy and remains the lowest-energy structure throughout the sampled volume range. The *Immm*, *Fm*-3*m*, and *P*4/*mmm* phases lie approximately 0.25–0.27 eV atom^−1^ above *Cmcm*, whereas *Fmmm* is approximately 0.35 eV atom^−1^ higher. The previously reported *Pm*-3*n* phase has the highest minimum energy, approximately 0.49 eV atom^−1^ above *Cmcm*. Thus, *Cmcm* is the lowest-energy structure among the six Re_3_Zr polymorphs considered at 0 K and 0 GPa. This fixed-composition comparison, however, does not by itself establish stability against decomposition into other Re–Zr phases.

To evaluate stability against decomposition, the formation enthalpy per atom of each ReₓZrᵧ compound was calculated according to

(2) $$\Delta H_{f}=\frac{E_{tot}\left({Re}_{x}{Zr}_{y}\right)-xE_{Re}^{hcp}-yE_{Zr}^{hcp}}{x+y}$$

where $E_{tot}\left({Re}_{x}{Zr}_{y}\right)$ is the calculated total energy of the compound, and $E_{Re}^{hcp}$ and $E_{Zr}^{hcp}$ are the calculated energies per atom of elemental Re and Zr in their respective hexagonal ground-state structures. Figure 2b presents a selected-phase partial convex hull constructed exclusively from energies calculated using the same computational settings and elemental reference states. The calculated formation enthalpies of ReZr_2_, ReZr, Re_2_Zr, and Re_24_Zr_5_ are −0.138, −0.216, −0.374, and −0.230 eV atom^−1^, respectively. For Re_2_Zr, the present value differs by 0.017 eV atom^−1^ from the Materials Project value of −0.357 eV atom^−1^ for the corresponding Re_2_Zr entry (mp-12109) [36]. This database value is used only as an external numerical reference and was not included in constructing Figure 2b. Re_25_Zr_21_ was excluded from the partial hull because an energy obtained using the same computational settings was unavailable. The Materials Project reports a formation energy of −0.347 eV atom^−1^ for Re_25_Zr_21_ (mp-574458) [37]; this value is likewise quoted only for reference and was not combined with the present DFT energies. The calculated formation enthalpy of *Pm-*3*n* Re_3_Zr, +0.255 eV atom^−1^, differs markedly from the negative value reported by Pan et al. [12]. All entries used in Figure 2b were evaluated within a single internally consistent reference scheme, and the present *Pm*-3*n* value is therefore retained. The origin of the discrepancy remains to be independently resolved.

At the Re_3_Zr composition, the relevant tie line of the selected-phase partial hull connects Re_2_Zr and Re_24_Zr_5_ and has an interpolated formation enthalpy of approximately −0.299 eV atom^−1^. The *Cmcm* structure has a formation enthalpy of −0.257 eV atom^−1^ and therefore lies approximately 0.042 eV atom^−1^ above this partial hull. Accordingly, *Cmcm* is not predicted to be a 0 K equilibrium ground-state phase, but it is the lowest-lying off-hull Re_3_Zr structure examined here and may be regarded as a metastable candidate subject to kinetic accessibility. Metastable phases can nevertheless be experimentally accessible when kinetic barriers hinder transformation to the equilibrium structure. For instance, metastable bixbyite-type V_2_O_3_ and δ-TaON were experimentally synthesized and shown to persist owing to kinetic stabilization [38,39]. These examples support the possible accessibility of low-lying metastable phases under suitable synthesis conditions. The *Immm* and *Fm*-3*m* structures have slightly negative formation enthalpies of −0.004 and −0.001 eV atom^−1^, respectively, but remain substantially above the partial hull. By contrast, the positive formation enthalpies of *Fmmm* (+0.091 eV atom^−1^), *P*4/*mmm* (+0.008 eV atom^−1^), and *Pm*-3*n* (+0.255 eV atom^−1^) indicate instability relative to the elemental reference states at 0 K. These thermodynamic classifications do not preclude the structures from being dynamically or mechanically stable local minima, which are assessed separately below.

**Figure 3:**
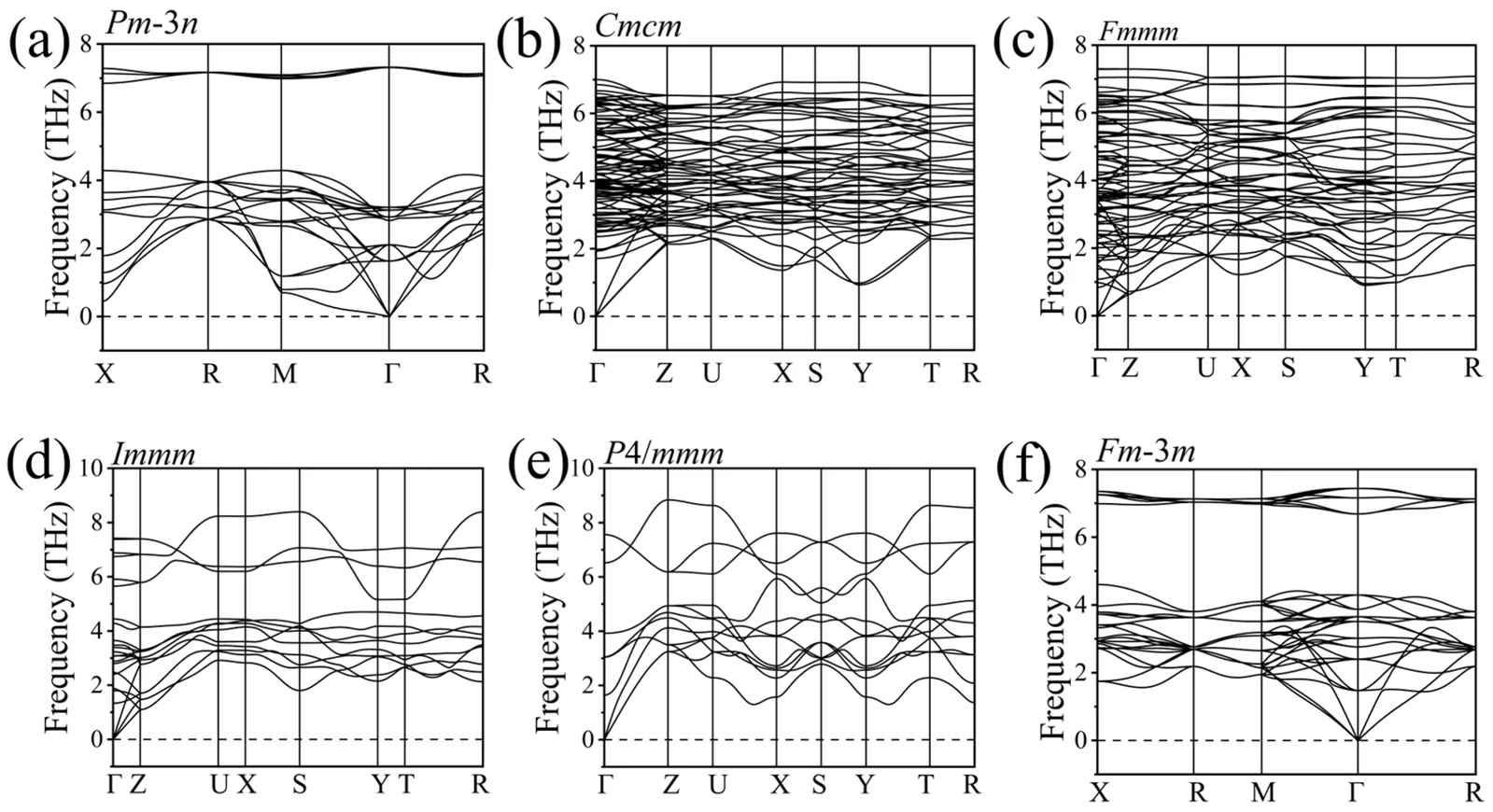
Calculated phonon dispersion relations of the six Re_3_Zr polymorphs at 0 K and ambient pressure: (**a**) *Pm*-3*n*, (**b**) *Cmcm*, (**c**) *Fmmm*, (**d**) *Immm*, (**e**) *P*4/*mmm*, and (**f**) *Fm*-3*m*.

Thermodynamic position relative to the partial convex hull and dynamical stability describe distinct aspects of phase stability. Phonon calculations were therefore used to determine whether the relaxed Re_3_Zr structures correspond to dynamically stable local minima. Figure 3 compares the phonon dispersion relations of all six Re_3_Zr polymorphs calculated using the phase-dependent supercells and consistent finite-displacement protocol described in Section 2. No imaginary frequencies are observed along the sampled high-symmetry paths, and the three acoustic branches approach zero frequency at the Γ point. All six relaxed structures can therefore be identified as dynamically stable local minima within the harmonic approximation at 0 K and ambient pressure. The phonon-branch distributions differ appreciably among the six polymorphs because of their distinct cell sizes and force-constant networks. The denser branch manifolds of *Cmcm* and *Fmmm* arise primarily from the larger numbers of atoms in their crystallographic cells. The *Immm* and *P*4/*mmm* spectra extend to approximately 8–9 THz, consistent with their comparatively stiff local bonding environments and narrow nearest-neighbor distance ranges. Both cubic structures, *Pm*-3*n* and *Fm*-3*m*, exhibit separated groups of high-frequency optical branches near 7 THz, although their lower-frequency branches show different dispersive behavior. These results establish the harmonic dynamical stability of the six structures as local minima, but do not alter their thermodynamic classification: in particular, the absence of imaginary phonon modes in *Pm*-3*n* does not offset its positive formation enthalpy, and the off-hull *Cmcm* structure remains a candidate metastable configuration rather than a 0 K equilibrium phase.

**Figure 4:**
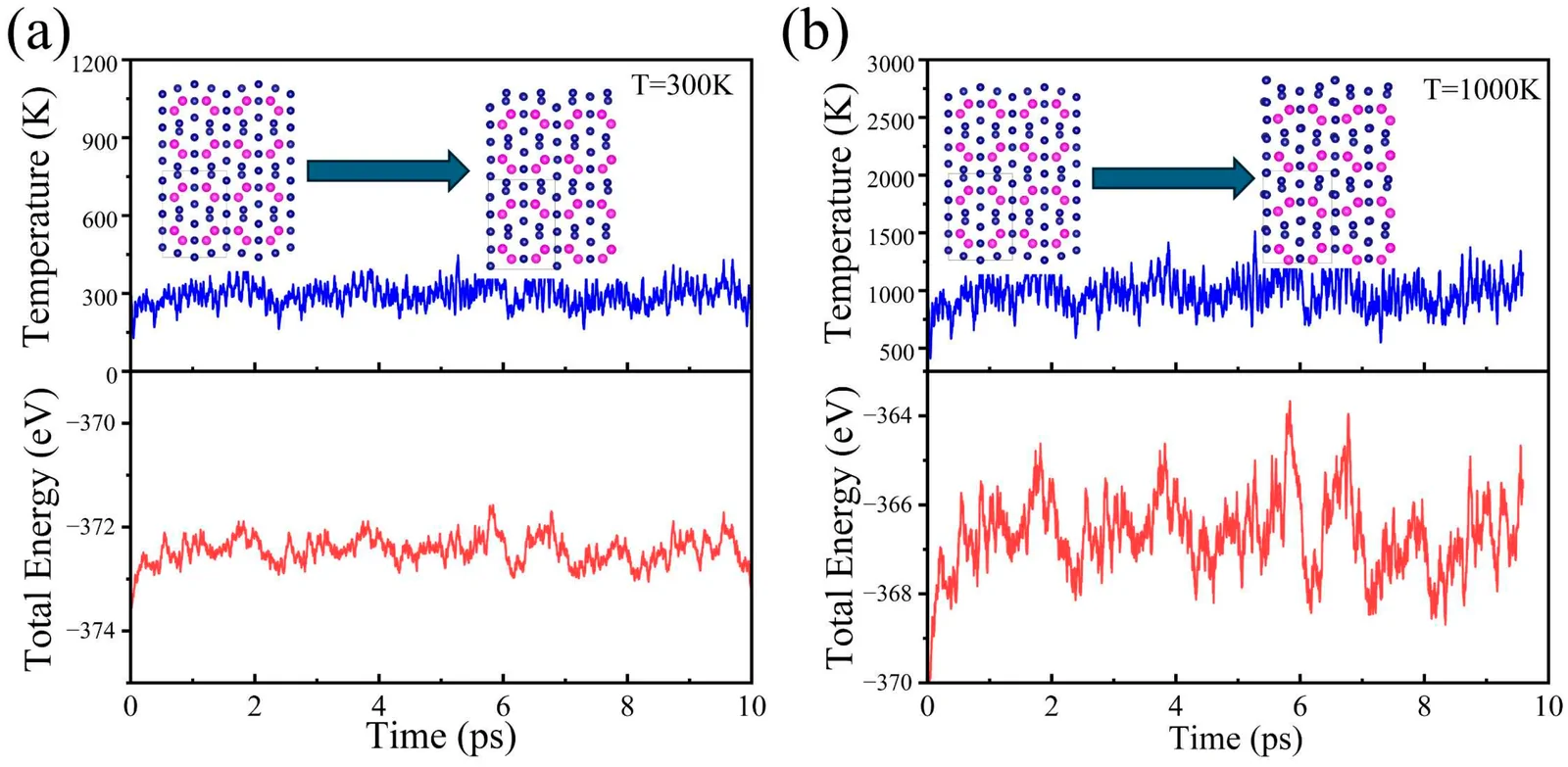
AIMD simulations of *Cmcm* at (**a**) 300 K and (**b**) 1000 K, showing the time evolution of instantaneous temperature (blue) and total energy (red) together with the initial and final structural configurations.

To further examine the finite-temperature structural persistence of the *Cmcm* phase, AIMD simulations were performed at 300 and 1000 K. As shown in Figure 4, the instantaneous temperatures fluctuate around the prescribed values, while after an initial equilibration period, the instantaneous temperatures fluctuate around the prescribed values, while the total energies exhibit bounded thermal fluctuations without sustained drift. The final configurations at both temperatures retain the overall framework of the initial *Cmcm* structure, although larger thermal displacements are observed at 1000 K. No obvious structural collapse or reconstruction is observed over the simulated time scales. These AIMD results therefore support the finite-temperature structural persistence of the *Cmcm* phase on the picosecond time scale.

Mechanical integrity under applied load is equally important for intermetallics because an elastic instability can trigger structural distortion before fracture or plastic flow. The second-order elastic constants quantify resistance to small strains and also underpin the modulus, anisotropy, and acoustic analyses presented later. Accordingly, mechanical stability was assessed from the *C_ij_* values listed in Table 3. Because the elastic tensors refer to fully relaxed structures at zero external pressure, the necessary and sufficient Born criteria for unstressed crystals were applied [40]. For the orthorhombic phases, these criteria are:

(3) $$C_{11}>0,C_{22}>0,C_{33}>0,C_{44}>0,C_{55}>0,C_{66}>0$$

(4) $$C_{ii}C_{jj}>C_{ij}^{2},\left(i,j\right)=\left(1,2\right),\left(1,3\right),\left(2,3\right)$$

(5) $$C_{11}C_{22}C_{33}+2C_{12}C_{23}C_{13}-C_{11}C_{23}^{2}-C_{22}C_{13}^{2}-C_{33}C_{12}^{2}>0$$

For the tetragonal *P*4/*mmm* phase, the corresponding stability conditions reduce to:

(6) $$C_{11}>\left|C_{12}\right|,2C_{13}^{2}<C_{33}\left(C_{11}+C_{12}\right),\;C_{44}>0,C_{66}>0$$

For the cubic *Pm*-3*n* and *Fm*-3*m* phases, they reduce further to:

(7) $$C_{11}-C_{12}>0,\;C_{11}+2C_{12}>0,\;C_{44}>0$$

Only the symmetry-independent elastic constants are reported in Table 3. For the tetragonal *P*4/*mmm* structure, symmetry requires $C_{22}\;$=${\;C}_{11}$, $C_{23}\;$=${\;C}_{13}$, and $C_{55\;}$=$\;C_{44}$. For the cubic *Pm*-3*n* and *Fm*-3*m* structures, $C_{11\;\;}$=${C\;}_{22}\;$=$\;C_{33}$, $C_{12}\;$=$\;C_{13\;}$=${\;C}_{23}$, and $C_{44}\;$=${\;C}_{55}\;$=${\;C}_{66}$. The remaining unlisted components are either symmetry-equivalent to the listed constants or vanish by symmetry.

Substitution of the calculated elastic constants into the corresponding Born criteria shows that all six structures satisfy the required inequalities and are therefore mechanically stable against infinitesimal homogeneous strains at 0 K and zero external pressure.

The reported *Pm*-3*n* phase [12] also serves as a benchmark for the elastic calculations. The present *C*_11_ and *C*_44_ values differ from the previous theoretical results by only 2.5% and 9.1%, respectively, whereas *C*_12_ is 18.3% larger. This level of agreement supports the reliability of the present stress–strain calculations. Although *Pm*-3*n* has the largest *C*_11_ value of 498 GPa, its much smaller *C*_44_ value of 36 GPa reveals weak resistance to the corresponding shear deformation. This contrast shows that high longitudinal stiffness does not necessarily imply strong resistance to shear. Similar shear softness is found for *Fmmm*, whose *C*_44_, *C*_55_, and *C*_66_ values are 59, 57, and 36 GPa, respectively. In contrast, *P*4/*mmm* exhibits the largest shear constants (*C*_44_ = 139 GPa and *C*_66_ = 120 GPa), while *Cmcm*, *Immm*, and *Fm*-3*m* also show substantially stronger shear resistance than the reference phase. Taken together with the energetic and phonon results, the elastic data demonstrate that the CALYPSO-predicted structures are mechanically stable at 0 K and ambient pressure and that several provide greater shear resistance than *Pm*-3*n*. These differences provide the basis for the subsequent polycrystalline modulus, ductility, anisotropy, and acoustic analyses.

The single-crystal elastic tensors were further converted into effective isotropic polycrystalline moduli using the Voigt–Reuss–Hill (VRH) approximation. The bulk modulus *B*, shear modulus *G*, and Young’s modulus *E* quantify the calculated small-strain resistance to volumetric, shear, and uniaxial deformation, respectively. These 0 K elastic averages are used here as comparative descriptors of the ideal structures; they do not include the effects of defects, grain boundaries, plastic deformation, temperature, or creep. Using the stiffness coefficients *C_ij_* and the elastic compliance coefficients *S_ij_*, where *S* = *C*^−1^, the Voigt upper bounds and Reuss lower bounds are expressed as [26,27,28]:

(8) $$B_{V}=\frac{1}{9}\left[C_{11}+C_{22}+C_{33}+2\left(C_{12}+C_{13}+C_{23}\right)\right]$$

(9) $$G_{V}=\frac{1}{15}\left[C_{11}+C_{22}+C_{33}-\left(C_{12}+C_{13}+C_{23}\right)+3\left(C_{44}+C_{55}+C_{66}\right)\right]$$

(10) $${B_{R}=\left[S_{11}+S_{22}+S_{33}+2\left(S_{12}+S_{13}+S_{23}\right)\right]}^{-1}$$

(11) $$G_{R}=15{\left[4\left(S_{11}+S_{22}+S_{33}\right)-4\left(S_{12}+S_{13}+S_{23}\right)+3\left(S_{44}+S_{55}+S_{66}\right)\right]}^{-1}$$

The effective bulk and shear moduli are taken as the arithmetic means of the corresponding bounds, and Young’s modulus, Poisson’s ratio, and the modulus ratio *k* are then obtained from:

(12) $$B=\frac{B_{V}+B_{R}}{2},\;\;G=\frac{G_{V}+G_{R}}{2}$$

(13) $$E=\frac{9BG}{3B+G},\;\;\nu =\frac{3B-2G}{2\left(3B+G\right)},\;\;k=\frac{G}{B}$$

Elastic anisotropy was quantified using the universal index *A^U^* [29], for which *A^U^* = 0 represents an elastically isotropic solid. The Chen and Tian models [30,31] were used to obtain empirical Vickers-hardness estimates from the calculated elastic moduli:

(14) $$A^{U}=5\frac{G_{V}}{G_{R}}+\frac{B_{V}}{B_{R}}-6.$$

(15) $$H_{v}^{C}=2{\left(k^{2}G\right)}^{0.585}-3$$

(16) $$H_{v}^{T}=0.92k^{1.137}G^{0.708}$$

Here, the subscripts *V* and *R* denote the Voigt and Reuss bounds, respectively, and *k* = *G*/*B*. The calculated macroscopic properties are summarized in Table 4. Because these models are empirical correlations based primarily on elastic moduli, they do not explicitly describe indentation-induced plasticity, defects, microstructure, or fracture. The resulting values are therefore interpreted only as comparative hardness indicators rather than direct predictions of experimental indentation hardness.

The universal anisotropy index reveals a clear contrast between the CALYPSO-predicted phases and the reference structure. *Fm*-3*m* is essentially isotropic (*A^U^* = 0.02), whereas *Pm*-3*n* exhibits a markedly larger *A^U^* of 3.53; the remaining predicted phases lie between these limits and retain comparatively weak-to-moderate anisotropy. The comparatively large anisotropy of *Pm*-3*n* is consistent with its low $C_{44}$ and indicates a pronounced directional dependence of its small-strain shear response. The contrast between *Pm*-3*n* and *Fm-3m* also shows that cubic symmetry alone does not imply elastic isotropy; the directional response must be determined from the elastic constants.

Although the absolute hardness values are model dependent, the Chen and Tian models reproduce the same trend: hardness increases with shear stiffness rather than with resistance to volumetric compression. *P*4/*mmm* gives the largest estimate of 10–11 GPa, consistent with its maximum *G*, whereas the shear-soft *Fmmm* and *Pm*-3*n* phases are the least hard. Thus, no single structure optimizes every mechanical attribute. Among the six phases, *Cmcm* combines the lowest static energy with high incompressibility, ductility, and low anisotropy; *P4/mmm* has the largest stiffness and hardness estimates, whereas *Immm* and *Fm-3m* combine high shear rigidity with low elastic anisotropy. These results describe the relative 0 K elastic responses of ideal crystals and should not be interpreted as measured hardness, ductility, or finite-temperature mechanical performance.

### 3.3. Elastic Anisotropy

Elastic anisotropy was examined to resolve the directional variation in the calculated small-strain response of the six Re_3_Zr structures. The Voigt–Reuss–Hill moduli describe an effective isotropic polycrystalline average but do not identify the stiffest and most compliant crystallographic directions. The directional bulk modulus *B*, Young’s modulus *E*, shear modulus *G*, and Poisson’s ratio *ν* were therefore evaluated from the elastic compliance tensor as follows [42]:

(17) $$B\left(\theta ,\varphi \right)={\left(S_{ijkk}n_{i}n_{j}\right)}^{-1}$$

(18) $$E\left(\theta ,\varphi \right)={{(S}_{ijkl}n_{i}n_{j}n_{k}n_{l})}^{-1}$$

(19) $$G\left(\theta ,\varphi ,\psi \right)={{(4S}_{ijkl}n_{i}m_{j}n_{k}m_{l})}^{-1}$$

(20) $$v\left(\theta ,\varphi ,\psi \right)=-\frac{S_{ijkl}n_{i}n_{j}m_{k}m_{l}}{S_{ijkl}n_{i}n_{j}n_{k}n_{l}}$$

Here, *S_ijkl_* is the fourth-rank elastic compliance tensor, and *n* and *m* are mutually orthogonal unit vectors specifying the loading and transverse directions, respectively. The angles *θ* and *φ* define *n*, whereas *ψ* describes the rotation of *m* about *n*. Because *B* and *E* depend only on *n*, they can be represented directly as three-dimensional surfaces. *G* and *ν* additionally depend on *m* and were therefore visualized using averages of their maximum and minimum values for each loading direction:

(21) $$\bar{G}\left(\theta ,\varphi \right)=\frac{G_{max}\left(\theta ,\varphi \right)+G_{min}\left(\theta ,\varphi \right)}{2}$$

(22) $$\bar{v}\left(\theta ,\varphi \right)=\frac{v_{max}\left(\theta ,\varphi \right)+v_{min}\left(\theta ,\varphi \right)}{2}$$

**Figure 5:**
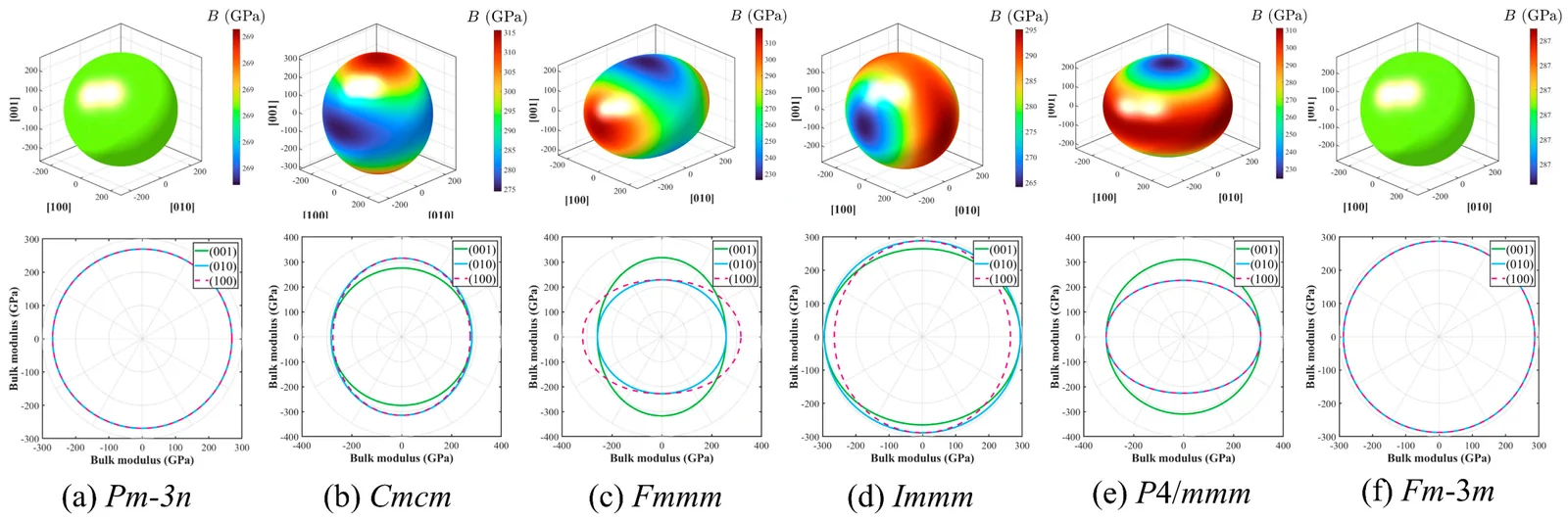
Three−dimensional surfaces and corresponding two-dimensional projections of the directional bulk modulus *B* for the six Re_3_Zr phases: (**a**) *Pm*-3*n*, (**b**) *Cmcm*, (**c**) *Fmmm*, (**d**) *Immm*, (**e**) *P4/mmm*, and (**f**) *Fm-3m*.

**Figure 6:**
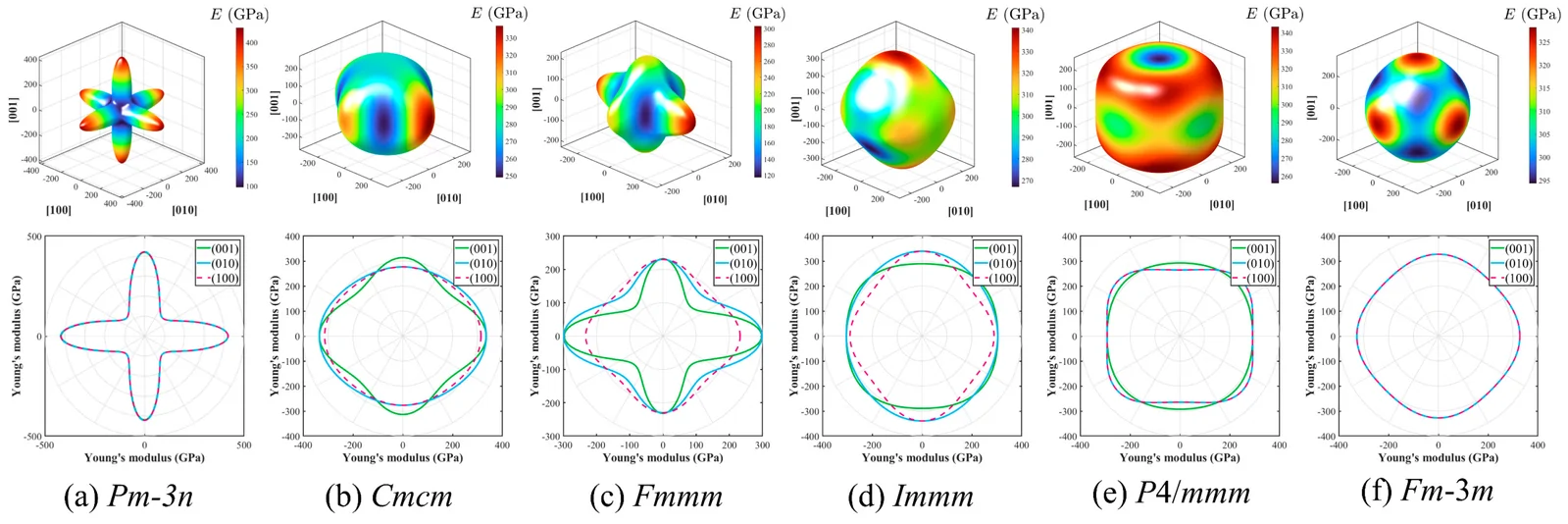
Three−dimensional surfaces and corresponding two-dimensional projections of the directional Young’s modulus *E* for the six Re_3_Zr phases: (**a**) *Pm*-3*n*, (**b**) *Cmcm*, (**c**) *Fmmm*, (**d**) *Immm*, (**e**) *P4/mmm*, and (**f**) *Fm-3m*.

**Figure 7:**
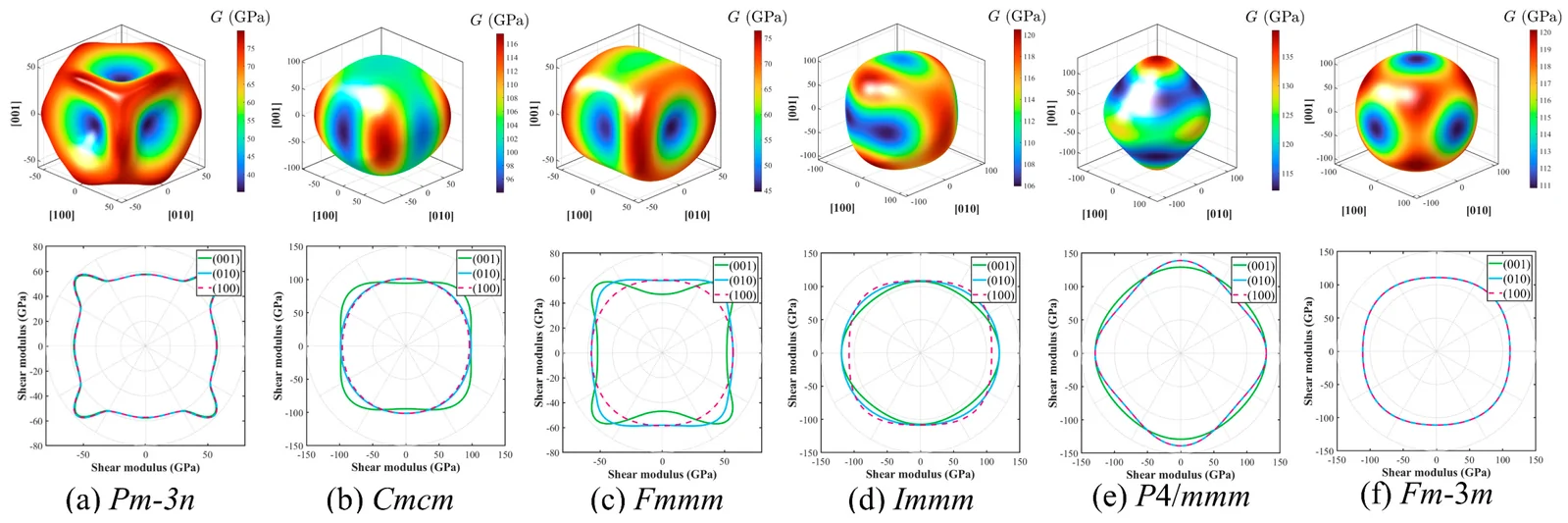
Three−dimensional surfaces and corresponding two-dimensional projections of the directional shear modulus *G* for the six Re_3_Zr phases: (**a**) *Pm*-3*n*, (**b**) *Cmcm*, (**c**) *Fmmm*, (**d**) *Immm*, (**e**) *P4/mmm*, and (**f**) *Fm-3m*.

**Figure 8:**
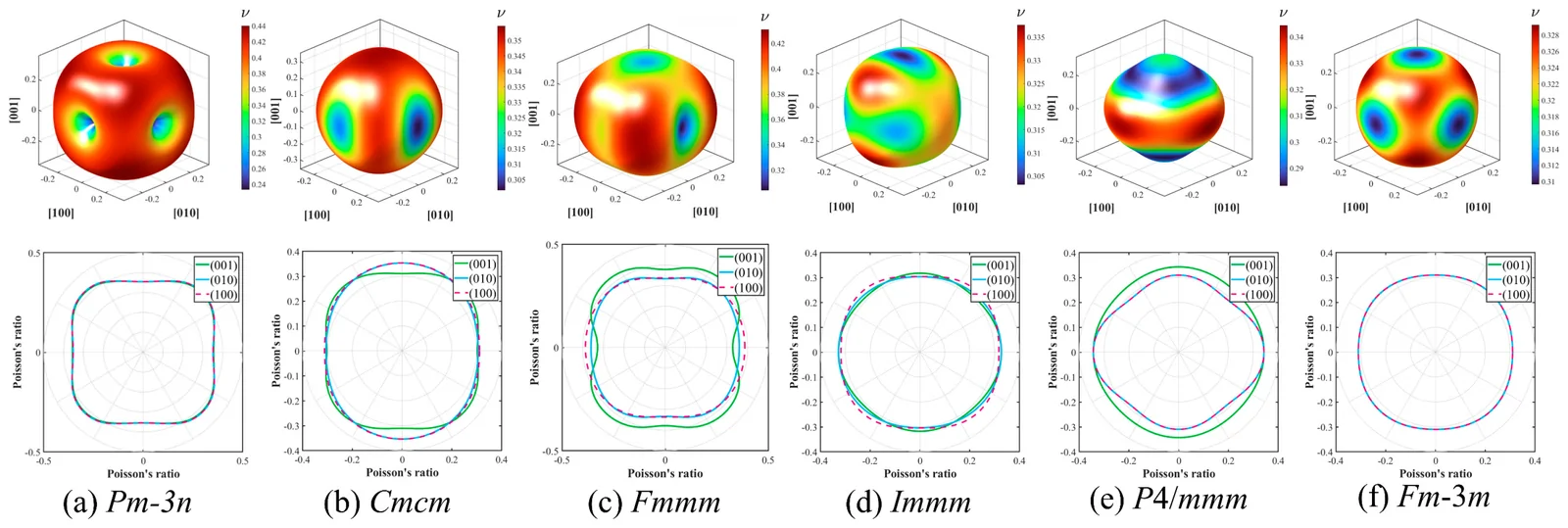
Three−dimensional surfaces and corresponding two-dimensional projections of Poisson’s ratio *ν* for the six Re_3_Zr phases: (**a**) *Pm*-3*n*, (**b**) *Cmcm*, (**c**) *Fmmm*, (**d**) *Immm*, (**e**) *P4/mmm*, and (**f**) *Fm-3m*.

A spherical surface with coincident circular projections represents an isotropic response; increasing distortion and separation among the (001), (010), and (100) projections indicate stronger directional dependence. In Figure 5, Figure 6 and Figure 7, red and blue regions denote larger and smaller directional moduli, respectively. In Figure 8, the colors indicate only larger or smaller Poisson’s-ratio values and should not be interpreted as stronger or weaker directional stiffness.

The bulk-modulus surfaces in Figure 5 show that similar polycrystalline *B* values can conceal different directional compression responses. *Pm*-3*n* and *Fm-3m* have nearly spherical surfaces and coincident projections, indicating almost direction-independent resistance to hydrostatic compression in this representation. By contrast, *Cmcm* and *Immm* show modest distortions, while *Fmmm* and *P4/mmm* exhibit clearer axial–basal differences; *P4/mmm* is more compressible along [001] than within the basal plane. The comparable *B* surfaces of *Pm*-3*n* and *Fm-3m* do not imply comparable overall elastic isotropy because their *E* and *G* surfaces differ strongly, as discussed below.

The six-phase comparison in Figure 6 and Figure 7 reveals a much wider variation in tensile and shear anisotropy than in bulk compression. *Pm*-3*n* exhibits the most distorted *E* and *G* surfaces, with sharp lobes and deep minima along selected directions. *Fmmm* shows the next strongest directional contrast, whereas *Cmcm*, *Immm*, and *P4/mmm* exhibit moderate-to-weak distortions. *Fm-3m* has the most regular surfaces and nearly overlapping projections, indicating the most uniform tensile and shear responses. The opposite behavior of the two cubic phases, *Pm*-3*n* and *Fm-3m*, demonstrates that cubic symmetry does not by itself guarantee elastic isotropy. The corresponding directional extrema and anisotropy ratios are summarized in Table 5.

Table 5 quantifies these trends. *Pm*-3*n* has by far the largest directional ratios, *E*_max_/*E*_min_ = 4.07 and *G*_max_/*G*_min_ = 4.72, because high maxima (424 and 171 GPa) coexist with low minima (104 and 36 GPa). *Fmmm* is the second-most anisotropic, with ratios of 2.47 and 2.96. In contrast, *Fm-3m* has the smallest ratios (1.11 and 1.13), followed by *Immm* (1.26 and 1.29); *Cmcm* and *P4/mmm* also remain within moderate ranges. Thus, the ranking of directional anisotropy agrees with *A^U^* and identifies compliant directions that are obscured by the polycrystalline averages. The corresponding Poisson’s-ratio surfaces are shown in Figure 8.

All six Poisson’s-ratio surfaces in Figure 8 remain positive; therefore, no auxetic response is obtained for the sampled loading and transverse directions. *Pm*-3*n* and *Fmmm* show the broadest directional variations and the largest local *ν* values, consistent with their pronounced shear compliance and anisotropy. *Cmcm* and *P4/mmm* show intermediate directional contrast, whereas *Immm* and *Fm-3m* have the narrowest variations. Taken together with the static energetic, mechanical, and dynamical results, the newly predicted structures should be regarded as possible candidates for further finite-temperature investigation.

### 3.4. Sound Velocities and Elastic Debye Temperatures

Sound velocities and elastic Debye temperatures were derived to compare the acoustic stiffness scales of the six Re_3_Zr structures. Within the isotropic elastic approximation, the sound velocities reflect the ratio between elastic stiffness and density, while the Debye temperature derived from the average sound velocity provides a characteristic elastic–acoustic energy scale. These quantities are comparative descriptors and do not constitute direct calculations of phonon lifetimes, lattice thermal conductivity, thermal expansion, heat capacity, anharmonicity, or finite-temperature phase stability.

Within the isotropic polycrystalline approximation, the longitudinal sound velocity *v_l_*, transverse sound velocity *v_t_*, and average sound velocity *v_m_* were calculated from the Voigt–Reuss–Hill bulk modulus *B*, shear modulus *G*, and density *ρ* using [32]:

(23) $$v_{l}=\sqrt{\frac{B+\frac{4}{3}G}{\rho }},\;v_{t}=\sqrt{\frac{G}{\rho }},v_{m}={\left[\frac{1}{3}\left(\frac{2}{{v_{t}}^{3}}+\frac{1}{{v_{l}}^{3}}\right)\right]}^{-\frac{1}{3}}$$

The Debye temperature was obtained from the average sound velocity according to [32]:

(24) $$\Theta_{D}=\;\frac{h}{k_{B}}{\left(\frac{3n}{4\pi V}\right)}^{\frac{1}{3}}\;v_{m}$$

Here, *h* and *k*_B_ are the Planck and Boltzmann constants, respectively, while *n* and *V* denote the number of atoms and volume of the selected unit cell; thus, *n*/*V* is the atomic number density. The resulting sound velocities are listed in Table 6.

**Figure 9:**
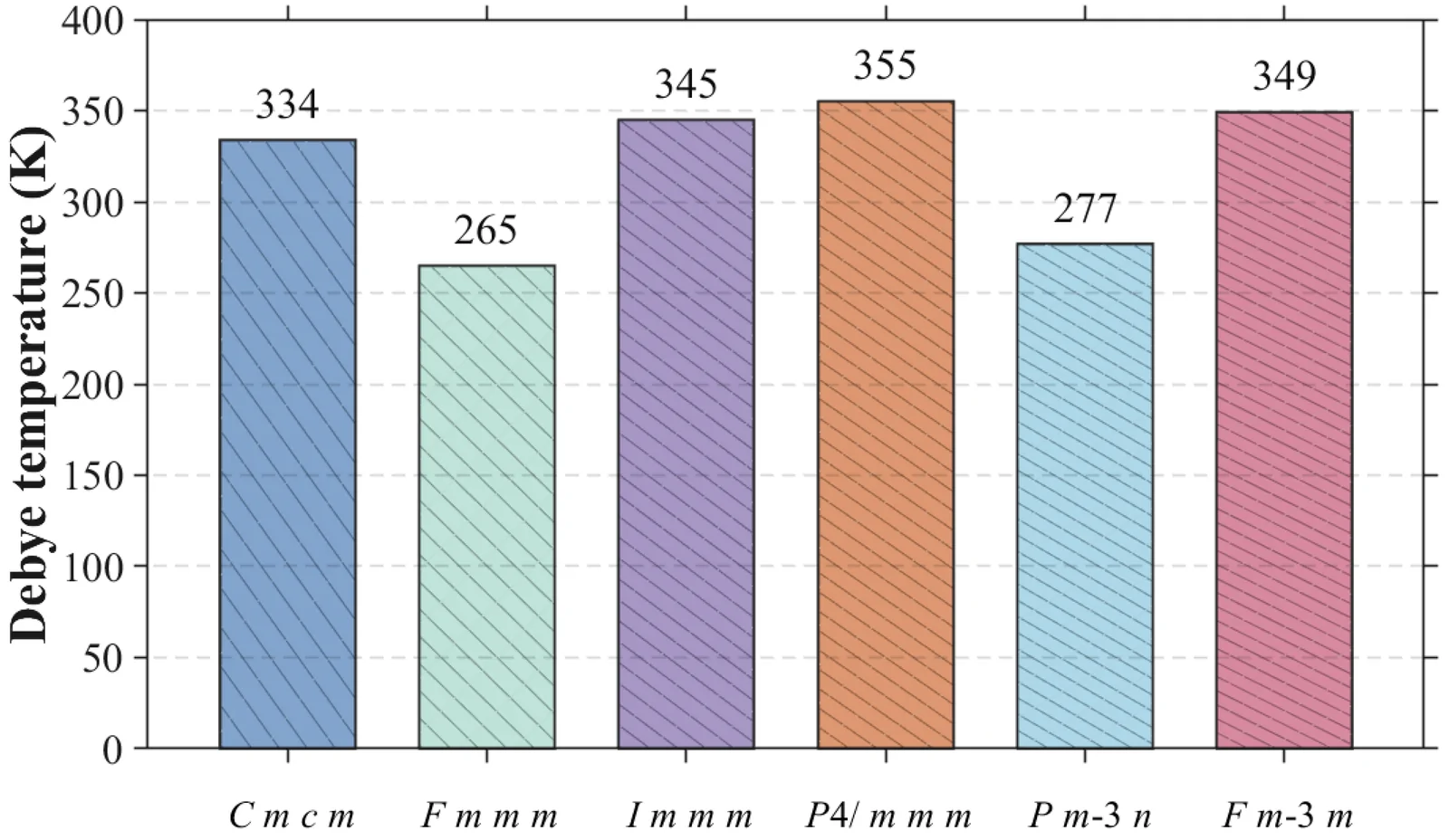
Calculated Debye temperatures *Θ_D_* of the six Re_3_Zr polymorphs.

The longitudinal velocities occupy a relatively narrow interval, whereas *v_t_* and *v_m_* distinguish the phases more clearly because they are governed primarily by shear rigidity. *P*4/*mmm* has the largest *v_t_* and *v_m_* values of 2713 and 3033 m s^−1^, respectively, followed closely by *Fm*-3*m* and *Immm*. This ordering is consistent with comparatively large shear moduli. *Cmcm* exhibits the highest *v_l_* of 5188 m s^−1^, reflecting its strong resistance to volumetric compression, while retaining an intermediate-to-high *v_m_* of 2878 m s^−1^. By contrast, *Fmmm* and the reference *Pm*-3*n* phase have substantially smaller transverse and average velocities, consistent with their low shear stiffness. Since the densities of these Re-rich phases vary only modestly, the velocity differences are controlled mainly by their elastic moduli rather than by mass density. The resulting Debye temperatures are shown in Figure 9.

As shown in Figure 9, the Debye temperatures follow the same sequence as *v_m_*, confirming that their differences originate predominantly from lattice stiffness. *P*4/*mmm* has the highest *Θ_D_* of 355 K, followed by *Fm*-3*m* (349 K), *Immm* (345 K), and *Cmcm* (334 K). The lower values of *Pm*-3*n* (277 K) and *Fmmm* (265 K) reflect their softer shear responses and lower characteristic phonon frequencies. Notably, four of the five CALYPSO-predicted phases have higher *Θ_D_* values than the reported *Pm*-3*n* structure, with the *P*4/*mmm* value being approximately 28% larger. Thus, *P*4/*mmm* provides the strongest overall elastic–vibrational rigidity, whereas *Fm*-3*m* and *Immm* combine high sound velocities with similarly elevated Debye temperatures. These elastic–vibrational trends provide a macroscopic basis for the electronic-structure analysis used subsequently to clarify their bonding origin.

### 3.5. Electronic Structure and Bonding Characteristics

**Figure 10:**
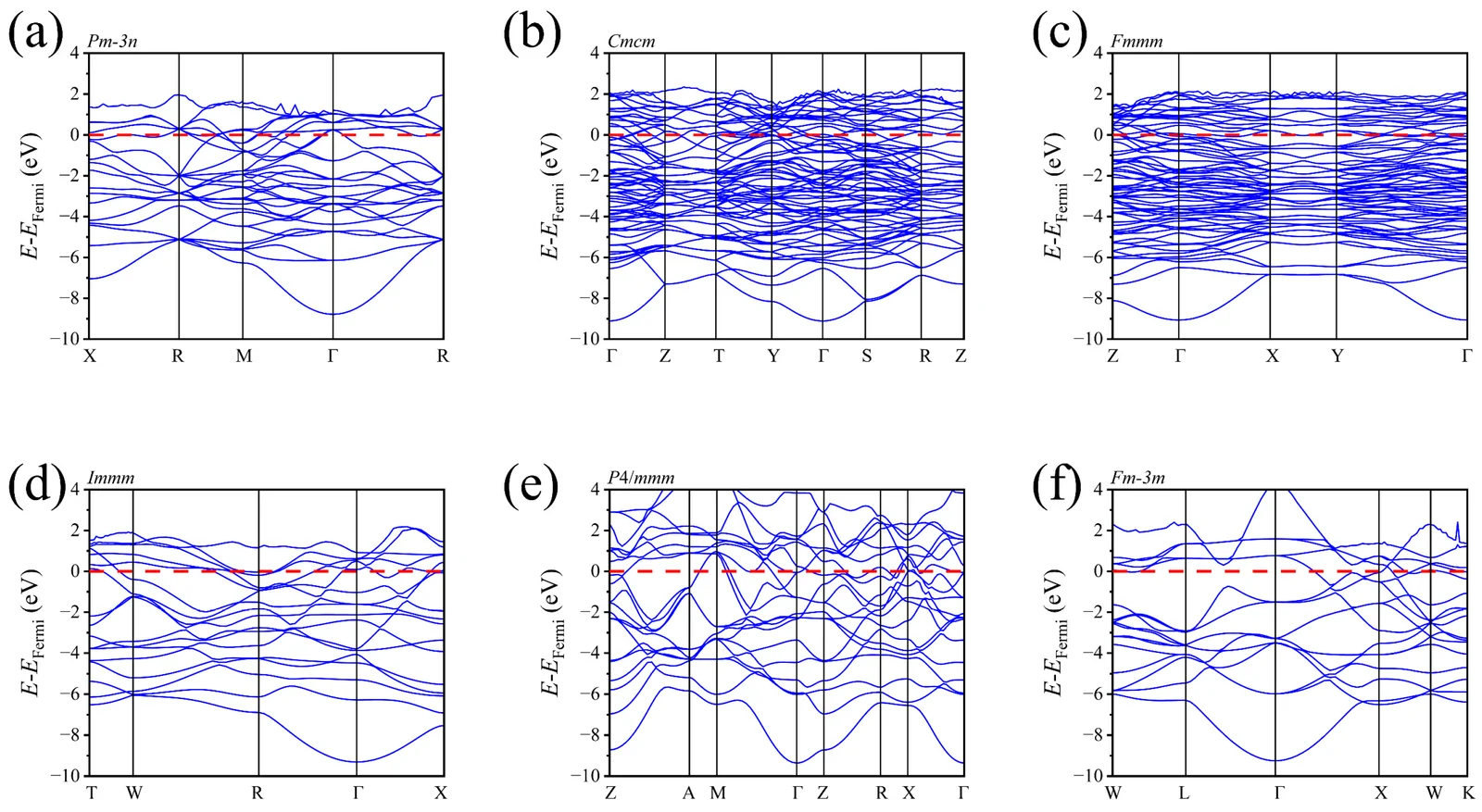
Electronic band structures of the six Re_3_Zr polymorphs: (**a**) *Pm*-3*n*, (**b**) *Cmcm*, (**c**) *Fmmm*, (**d**) *Immm*, (**e**) *P*4/*mmm*, and (**f**) *Fm*-3*m*. The Fermi level is set to 0 eV and indicated by the red dashed line.

**Figure 11:**
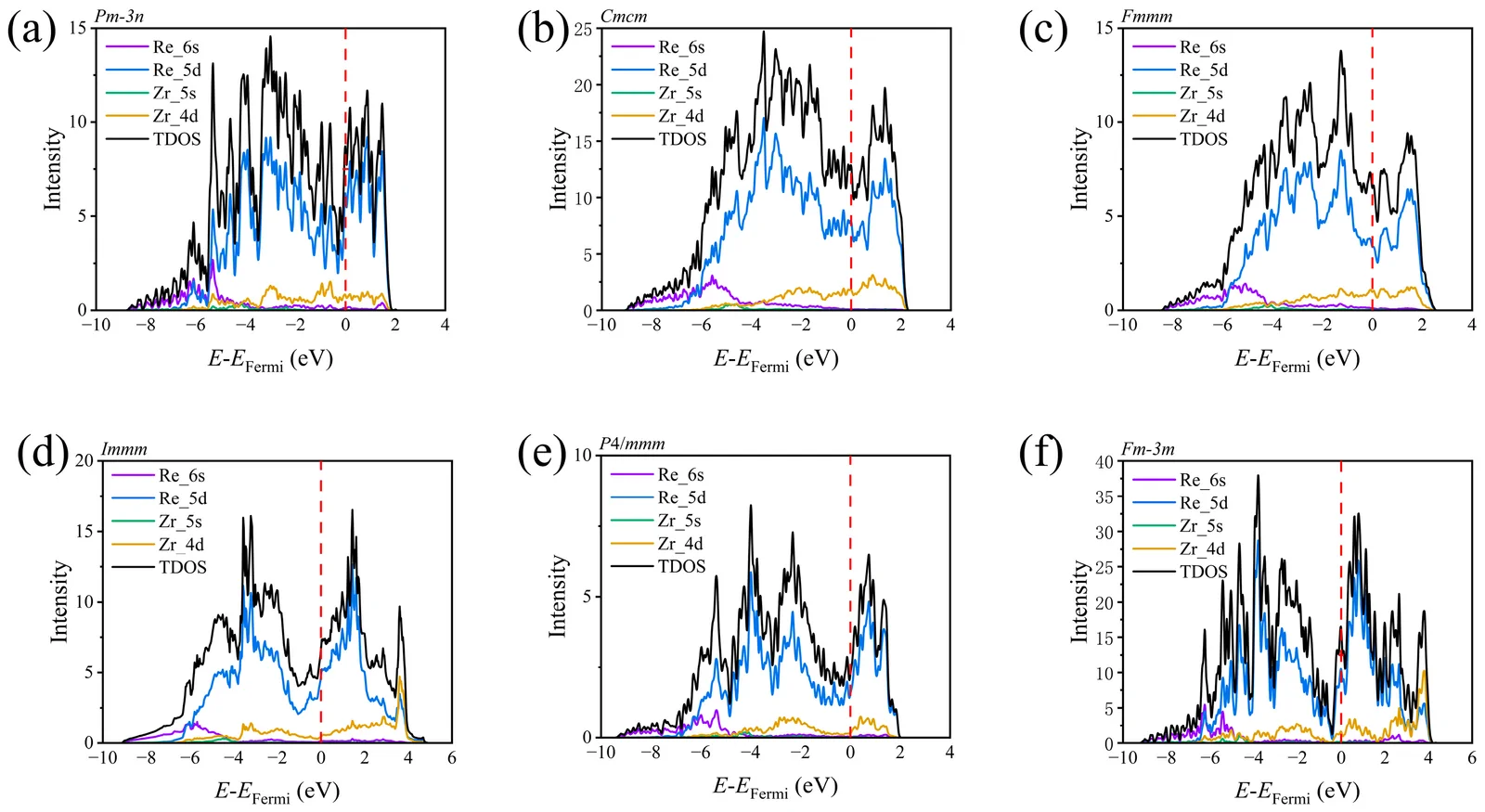
Total and orbital-projected densities of states of the six Re_3_Zr polymorphs: (**a**) *Pm*-3*n*, (**b**) *Cmcm*, (**c**) *Fmmm*, (**d**) *Immm*, (**e**) *P*4/*mmm*, and (**f**) *Fm*-3*m*. The Re-6*s*, Re-5*d*, Zr-5*s*, and Zr-4*d* contributions are shown; the Fermi level is set to 0 eV and marked by the red dashed line.

**Figure 12:**
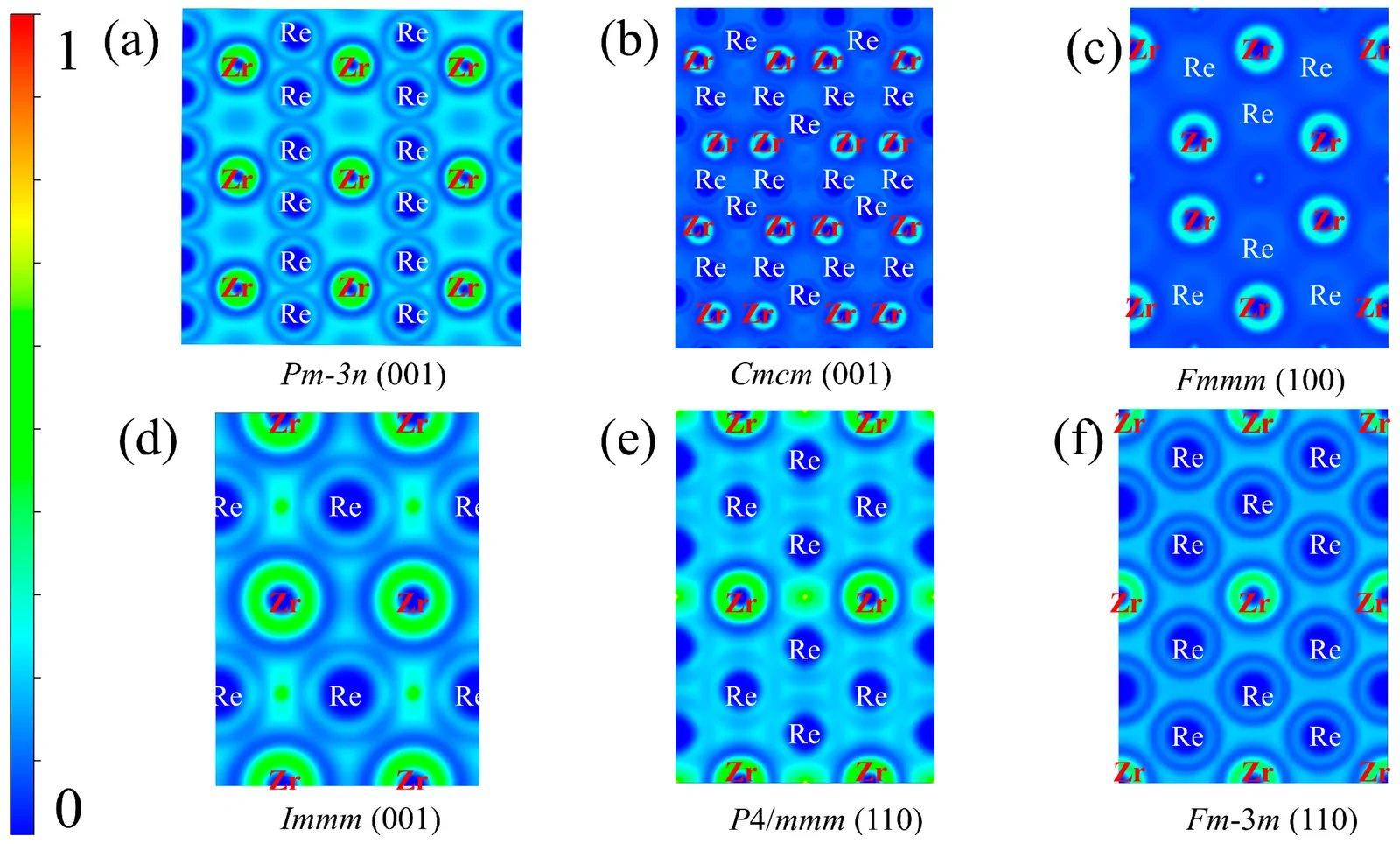
Two-dimensional electron localization function maps of the six Re_3_Zr polymorphs on representative crystallographic planes: (**a**) *Pm*-3*n* (001), (**b**) *Cmcm* (001), (**c**) *Fmmm* (100), (**d**) *Immm* (001), (**e**) *P*4/*mmm* (110), and (**f**) *Fm*-3*m* (110). The ELF scale ranges from 0 to 1.

Electronic properties provide the microscopic link between crystal structure and the mechanical and thermal responses discussed above. The band structures, total and orbital-projected densities of states, and electron localization functions were examined to identify the electronic origin of the phase-dependent properties of Re_3_Zr. The calculated band structures, densities of states, and electron localization maps are shown in Figure 10, Figure 11 and Figure 12, respectively.

As shown in Figure 10 and Figure 11, multiple bands cross the Fermi level *E_F_* in every phase, and the corresponding total density of states remains finite at *E_F_*, consistently confirming the metallic character of all six Re_3_Zr structures. The projected density of states further shows that the states around *E_F_* are dominated by Re-5d orbitals, with a smaller but non-negligible contribution from Zr-4d orbitals. The broad energetic overlap between the Re-5d and Zr-4d states throughout the valence region and near *E_F_* is consistent with appreciable Re–Zr d–d hybridization, whereas the Re-6s states are concentrated mainly at lower energies and the Zr-5s contribution remains weak over the displayed energy range. Despite differences in the detailed band dispersions and density-of-states profiles among the polymorphs, they share the same essential electronic features: Re-5d-dominated metallic states at *E_F_* and appreciable Re–Zr d–d hybridization.

The bonding nature was further characterized using the electron localization function (ELF) [33], as shown in Figure 12. In all six phases, the interatomic regions are dominated by low ELF values (blue–cyan), with no continuous high-ELF basins between Re and Zr. Together with the bands crossing E*_F_* and the finite density of states at E*_F_*, this feature indicates predominantly delocalized metallic bonding in Re_3_Zr. The enhanced ELF around Zr is largely atom-centered and does not indicate strong two-center Re–Zr covalent bonding. *Fmmm* exhibits the weakest interstitial localization, consistent with its low shear modulus and hardness. By comparison, localized ELF maxima at selected interatomic sites in *Immm* and *P4/mmm* suggest a modest directional bonding component, in agreement with their higher shear stiffness. The relatively uniform ELF distribution in *Fm−3m* is also consistent with its nearly isotropic elastic response. Overall, structural symmetry modifies the spatial localization of valence electrons without changing the predominantly metallic bonding framework.

**Figure 13:**
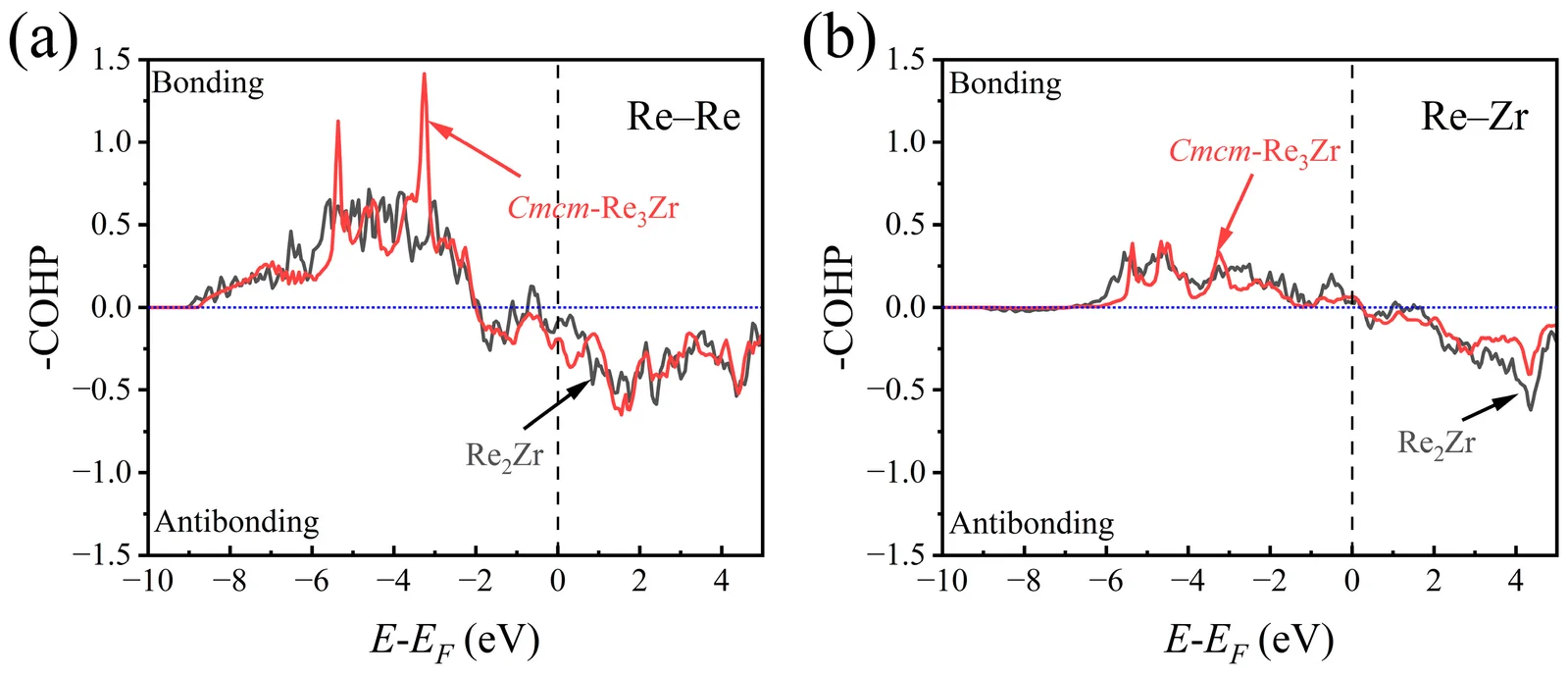
Energy-resolved −COHP curves for representative Re–Re and Re–Zr interactions in *Cmcm* and Re_2_Zr. (**a**) Re–Re: Re9–Re11 (2.560 Å) and Re7–Re10 (2.566 Å). (**b**) Re–Zr: Zr1–Re28 (2.887 Å) and Zr4–Re12 (3.100 Å). Positive and negative −COHP values indicate bonding and antibonding states, respectively; the black dashed line marks the Fermi level.

To complement the qualitative ELF analysis, the bonding interactions in *Cmcm* were further quantified using COHP and −ICOHP analysis and compared with those in Re_2_Zr. As summarized in Table 7, the strongest Re–Re interaction in *Cmcm* has a −ICOHP value of 2.18 eV bond^−1^ at 2.560 Å, comparable to 2.11 eV bond^−1^ at 2.566 Å in Re_2_Zr. The short Re–Zr interaction in *Cmcm* reaches 1.63 eV bond^−1^ at 2.887 Å, whereas the maximum value in Re_2_Zr is approximately 1.09 eV bond^−1^. The corresponding −COHP curves in Figure 13 show substantial bonding contributions below the Fermi level, with partial antibonding contributions near *E_F_*. These results reveal appreciable local Re–Re and Re–Zr bonding contributions in *Cmcm* while remaining consistent with the predominantly delocalized metallic bonding picture inferred from the ELF and electronic structures.

## 4. Conclusions

A CALYPSO global structure search combined with first-principles calculations identified five previously unreported hypothetical Re_3_Zr structures with *Cmcm*, *Fmmm*, *Immm*, *P*4/*mmm*, and *Fm*-3*m* symmetries and compared them consistently with the proposed *Pm*-3*n* phase. All five CALYPSO structures lie below *Pm*-3*n* in fixed-composition energy, and *Cmcm* is the lowest-energy polymorph at 0 K and 0 GPa. Its formation enthalpy is −0.257 eV atom^−1^, approximately 0.042 eV atom^−1^ above the selected-phase partial hull. The *Cmcm* structure is therefore classified as a low-lying off-hull metastable candidate rather than a 0 K equilibrium ground-state phase. *Immm* and *Fm*-3*m* have slightly negative formation enthalpies but remain well above the partial hull, whereas *Fmmm*, *P*4/*mmm*, and *Pm*-3*n* have positive formation enthalpies and are unstable relative to the elemental reference states at 0 K.

The phonon dispersions and elastic constants show that all six structures are dynamically and mechanically stable local minima at ambient pressure. AIMD simulations further show that the *Cmcm* framework persists without obvious structural reconstruction at 300 and 1000 K over the simulated time scales. Their comparative mechanical and acoustic properties depend strongly on crystal symmetry. *Cmcm* combines high resistance to volume compression with ductility indicators and relatively weak anisotropy. *P*4/*mmm* has the largest tensile and shear stiffnesses, Chen–Tian hardness estimates, average sound velocity, and elastic Debye temperature, while *Fm*-3*m* exhibits the most uniform directional elasticity. All six polymorphs are metallic, with Re-5d-dominated states near the Fermi level and appreciable Re-5d–Zr-4d hybridization. For *Cmcm*, the additional COHP analysis identifies pronounced Re–Re and short Re–Zr bonding contributions, while remaining consistent with the predominantly delocalized metallic bonding picture. These results establish a consistent 0 K structural, thermodynamic, vibrational, mechanical, and electronic reference for subsequent experimental searches and finite-temperature or kinetic assessments of Re_3_Zr.

## Figures and Tables

**Table 1:** Calculated lattice parameters *a*, *b*, and *c* (Å), space-group number (SG No.), crystal system (Struc.), number of formula units (Z), unit-cell volume *V* (Å^3^), and density *ρ* (g cm^−3^) of the six Re_3_Zr phases, together with the available theoretical result.

Phase	SG No.	Struc.	a	b	c	Z	*V*	*ρ*	Ref.
*Cmcm*	63	Orth.	8.674	11.668	5.338	8	540.22	15.98	This work
*Fmmm*	69	Orth.	4.992	13.015	8.562	8	556.26	15.52	This work
*Immm*	71	Orth.	3.158	4.550	9.090	2	130.61	16.52	This work
*P*4/*mmm*	123	Tetra.	3.167		6.553	1	65.75	16.41	This work
*Pm*-3*n*	223	Cub.	5.113			2	133.64	16.15	This work
*Pm*-3*n*			5.116			2		16.12	Theo. [12]
*Fm*-3*m*	225	Cub.	6.384			4	260.15	16.59	This work

**Table 2:** Nonequivalent atomic sites, Wyckoff positions, coordination environments (CE), coordination numbers (CN), and nearest-neighbor bond-length ranges (Å) of the optimized Re_3_Zr phases.

Phase	Atom	Wyckoff Positions				CE	CN	Bond Length
		Site	x	y	z			
*Cmcm*	Zr1	8g	0.186	0.693	0.750	Zr_3_Re_13_	16	2.60–3.08
	Re4	8g	0.237	0.952	0.750	Zr_4_Re_9_	13	
	Re1	8f	0.000	0.115	0.510	Zr_4_Re_8_	12	
	Re6	4c	0.000	0.306	0.750	Zr_6_Re_6_	12	
	Re7	4b	0.000	0.500	1.000	Zr_4_Re_8_	12	
*Fmmm*	Zr1	8h	0.500	0.121	0.000	Zr_0_Re_12_	12	2.57–3.04
	Re1	8d	0.250	0.000	0.750	Zr_4_Re_4_	8	
	Re3	16m	0.000	0.826	0.846	Zr_4_Re_8_	12	
*Immm*	Zr1	2d	0.000	0.500	0.000	Zr_0_Re_8_	8	2.72–2.82
	Re1	4i	0.000	0.000	0.244	Zr_2_Re_6_	8	
	Re2	2b	0.500	0.000	0.000	Zr_4_Re_4_	8	
*P*4/*mmm*	Zr1	1c	0.500	0.500	0.000	Zr_0_Re_8_	8	2.72–2.83
	Re1	2g	0.000	0.000	0.265	Zr_4_Re_4_	8	
	Re3	1d	0.500	0.500	0.500	Zr_0_Re_8_	8	
*Pm*-3*n*	Zr1	2a	0.000	0.000	0.000	Zr_0_Re_12_	12	2.56–2.86
	Re1	6d	0.500	0.000	0.250	Zr_4_Re_2_	6	
*Fm*-3*m*	Zr1	4a	0.000	0.000	0.000	Zr_0_Re_8_	8	2.76–2.76
	Re1	4b	0.500	0.000	0.000	Zr_0_Re_8_	8	
	Re2	8c	0.750	0.750	0.250	Zr_4_Re_4_	8	

**Table 3:** Calculated second-order elastic constants *C*_ij_ (GPa) of the six Re_3_Zr phases, together with the available theoretical values for the *Pm*-3*n* phase.

Phase	*C*_11_	*C*_12_	*C*_13_	*C*_22_	*C*_23_	*C*_33_	*C*_44_	*C*_55_	*C*_66_
*Cmcm*	461	183	221	447	229	436	98	105	91
*Fmmm*	415	199	174	395	224	369	59	57	36
*Immm*	441	216	198	421	195	459	98	120	118
*P*4/*mmm*	435	217	202			383	139		120
*Pm*-3*n*	498	155					36		
*Pm*-3*n* [12]	511	131					33		
*Fm*-3*m*	454	204					111		

**Table 4:** Calculated polycrystalline bulk modulus *B*, Young’s modulus *E*, shear modulus *G*, Poisson’s ratio *ν*, Pugh ratio *B*/*G* [41], universal elastic anisotropy index *A^U^*, and Vickers hardness estimated using the Chen $H_{v\;\;}^{C}$ [30] and Tian $H_{v}^{T}\;$ [31] models for the six Re_3_Zr phases. Elastic moduli and hardness values are in GPa.

Phase	*B*	*E*	*G*	v	*B*/*G*	*A* ^U^	$H_{v}^{C}$	$H_{v}^{T}$
*Cmcm*	290	281	105	0.34	2.76	0.10	6	8
*Fmmm*	263	179	65	0.39	4.07	0.79	1	4
*Immm*	282	302	114	0.32	2.47	0.05	8	9
*P*4/*mmm*	277	316	121	0.31	2.29	0.10	10	11
*Pm*-3*n*	269	197	72	0.38	3.76	3.53	2	4
*Fm*-3*m*	287	308	117	0.32	2.47	0.02	8	10

**Table 5:** Calculated minimum and maximum directional Young’s moduli *E_min_* and *E_max_*, minimum and maximum directional shear moduli *G_min_* and *G_max_*, and the corresponding anisotropy ratios *E_max_*/*E_min_* and *G_max_*/*G_min_* for the six Re_3_Zr phases. Moduli are in GPa.

Phase	*E*_min_	*E*_max_	*E*_max_/*E*_min_	*G*_min_	*G*_max_	*G*_max_/*G*_min_
*Cmcm*	251	335	1.33	91	135	1.48
*Fmmm*	121	299	2.47	36	107	2.96
*Immm*	269	339	1.26	98	127	1.29
*P*4/*mmm*	258	342	1.33	103	139	1.35
*Pm*-3*n*	104	424	4.07	36	171	4.72
*Fm*-3*m*	295	328	1.11	111	125	1.13

**Table 6:** Calculated longitudinal ($v_{l}$), transverse ($v_{t}$), and average ($v_{m}$) sound velocities for the six Re_3_Zr phases. Velocities are in m s^−1^.

Phase	$v_{l}$	$v_{t}$	$v_{m}$
*Cmcm*	5188	2564	2878
*Fmmm*	4744	2041	2306
*Immm*	5127	2628	2943
*P*4/*mmm*	5164	2713	3033
*Pm*-3*n*	4751	2105	2376
*Fm*-3*m*	5165	2650	2968

**Table 7:** Comparison of the interatomic distances and −ICOHP values for Re–Re, Re–Zr, and Zr–Zr interactions in *Cmcm* and Re_2_Zr. The statistics include all non-zero interatomic contacts within the common 3.3 Å cutoff.

Phase	Interaction	No. of Contacts	Range (Å)	Mean (eV Bond^−1^)	Max (eV Bond^−1^)
*Cmcm*	Re–Re	96	2.560–3.076	1.472	2.182
	Re–Zr	104	2.887–3.228	1.051	1.627
	Zr–Zr	12	3.186–3.224	1.156	1.180
Re_2_Zr	Re–Re	24	2.566–2.722	1.675	2.114
	Re–Zr	48	3.087–3.108	1.057	1.085
	Zr–Zr	8	3.237–3.266	1.028	1.032

## Data Availability

The original contributions presented in this study are included in the article/supplementary material. Further inquiries can be directed to the corresponding authors.

## Supplementary Materials

The following supporting information can be downloaded at: https://www.mdpi.com/article/10.3390/ma19183844/s1, Tables S1–S6: Optimized crystal structures of the *Cmcm*, *Fmmm*, *Immm*, *P*4/*mmm*, *Pm*-3*n*, and *Fm*-3*m* Re_3_Zr polymorphs in VASP POSCAR format.
