# Supplementary material for: Crystal Structure Prediction and 0 K Stability Assessment of Hypothetical Re3Zr Polymorphs: Vibrational, Elastic, and Electronic Properties
Source: Materials (Basel). 2026 Sep 9;19(18):3844. doi: 10.3390/ma19183844 (PMC13608423; doi:10.3390/ma19183844)
Supplement: Supplementary file 1 [file materials-19-03844-s001.zip › materials-4475321-supplementary.pdf]

SUPPLEMENTARY MATERIALS

Crystal Structure Prediction and 0 K Stability Assessment of Hypothetical Re_3Zr Polymorphs: Vibrational, Elastic, and Electronic Properties

Mardan Mamatyusup, Qun Wei, Zhengzhe Lin, Jing Luo, and Meiguang Zhang

Contents. This document provides the fully optimized crystal structures of the six Re_3Zr polymorphs examined in the manuscript. Each structure is presented in VASP POSCAR format using the supplied scale factor, lattice vectors, elemental order, atom counts, and fractional coordinates.

Data note. The element order is Zr followed by Re, and all coordinates are given in Direct mode. Optional zero-velocity records following the coordinate blocks in the source files have been omitted because they are not part of the optimized crystal geometry. Each block below can be copied directly into a plain-text file named POSCAR.

Summary of the supplied structures

Entry	Phase	Space-group No.	Atoms/cell	Atom counts
Table S1	*Cmcm*	63	32	Zr 8; Re 24
Table S2	*Fmmm*	69	32	Zr 8; Re 24
Table S3	*Immm*	71	8	Zr 2; Re 6
Table S4	*P4/mmm*	123	4	Zr 1; Re 3
Table S5	*Pm-3n*	223	8	Zr 2; Re 6
Table S6	*Fm-3m*	225	16	Zr 4; Re 12

Table S1. Optimized VASP POSCAR for *Cmcm*-Re_3Zr.

Space group No. 63; 32 atoms per supplied cell; Direct fractional coordinates.

```
1:Cmcm(63)
   1.00000000000000
     8.6738080417990222    0.0000000000000000    0.0000000000000000
     0.0000000000000000   11.6675427297881313    0.0000000000000000
     0.0000000000000000    0.0000000000000000    5.3379923699114364
   Zr   Re
     8    24
Direct
  0.1858709419254147  0.6927205202662660  0.2500000000000000
  0.8141290580745854  0.6927205202662660  0.2500000000000000
  0.6858709419254146  0.8072794797337340  0.7500000000000000
  0.3141290580745853  0.8072794797337340  0.7500000000000000
  0.6858709419254146  0.1927205202662589  0.2500000000000000
  0.3141290580745853  0.1927205202662589  0.2500000000000000
  0.1858709419254147  0.3072794797337411  0.7500000000000000
  0.8141290580745854  0.3072794797337411  0.7500000000000000
 -0.0000000000000000  0.1145819808887081  0.0102308944856405
 -0.0000000000000000  0.8854180191112917  0.9897691055143597
 -0.0000000000000000  0.1145819808887081  0.4897691055143595
  0.2370295843644817  0.9519985984469270  0.2500000000000000
 -0.0000000000000000  0.8854180191112917  0.5102308944856403
 -0.0000000000000000  0.3057696680330703  0.2500000000000000
```

```
 -0.0000000000000000  0.5000000000000000  0.5000000000000000
  0.7629704156355185  0.9519985984469270  0.2500000000000000
  0.7629704156355185  0.0480014015530729  0.7500000000000000
  0.2370295843644817  0.0480014015530729  0.7500000000000000
 -0.0000000000000000  0.6942303319669296  0.7500000000000000
 -0.0000000000000000  0.5000000000000000  0.0000000000000000
  0.5000000000000000  0.6145819808887083  0.0102308944856405
  0.5000000000000000  0.3854180191112920  0.9897691055143597
  0.5000000000000000  0.6145819808887083  0.4897691055143595
  0.7370295843644815  0.4519985984469271  0.2500000000000000
  0.5000000000000000  0.3854180191112920  0.5102308944856403
  0.5000000000000000  0.8057696680330704  0.2500000000000000
  0.5000000000000000 -0.0000000000000000  0.5000000000000000
  0.2629704156355183  0.4519985984469271  0.2500000000000000
  0.2629704156355183  0.5480014015530730  0.7500000000000000
  0.7370295843644815  0.5480014015530730  0.7500000000000000
  0.5000000000000000  0.1942303319669297  0.7500000000000000
  0.5000000000000000 -0.0000000000000000  0.0000000000000000
```

Table S2. Optimized VASP POSCAR for *Fmmm*-Re_3Zr.

Space group No. 69; 32 atoms per supplied cell; Direct fractional coordinates.

```
10:Fmmm(69)
   1.00000000000000
     4.9920200187252028    0.0000000000000000    0.0000000000000000
     0.0000000000000000   13.0149064813633899    0.0000000000000000
     0.0000000000000000    0.0000000000000000    8.5617778423241955
   Zr   Re
     8    24
Direct
  0.5000000000000000  0.1214609731956032 -0.0000000000000000
  0.5000000000000000  0.8785390268043968 -0.0000000000000000
  0.5000000000000000  0.6214609731956032  0.5000000000000000
  0.5000000000000000  0.3785390268043968  0.5000000000000000
  0.0000000000000000  0.1214609731956032  0.5000000000000000
 -0.0000000000000000  0.8785390268043968  0.5000000000000000
  0.0000000000000000  0.6214609731956032 -0.0000000000000000
 -0.0000000000000000  0.3785390268043968 -0.0000000000000000
  0.2500000000000000  0.0000000000000000  0.7500000000000000
  0.7500000000000000  0.0000000000000000  0.7500000000000000
  0.0000000000000000  0.8256333157286304  0.8463143417908849
 -0.0000000000000000  0.1743666842713695  0.1536856582091151
 -0.0000000000000000  0.8256333157286304  0.1536856582091151
 -0.0000000000000000  0.1743666842713695  0.8463143417908849
  0.2500000000000000  0.5000000000000000  0.2500000000000000
  0.7500000000000000  0.5000000000000000  0.2500000000000000
 -0.0000000000000000  0.3256333157286305  0.3463143417908849
 -0.0000000000000000  0.6743666842713696  0.6536856582091151
 -0.0000000000000000  0.3256333157286305  0.6536856582091151
  0.0000000000000000  0.6743666842713696  0.3463143417908849
  0.7500000000000000  0.0000000000000000  0.2500000000000000
  0.2500000000000000  0.0000000000000000  0.2500000000000000
  0.5000000000000000  0.8256333157286304  0.3463143417908849
  0.5000000000000000  0.1743666842713695  0.6536856582091151
  0.5000000000000000  0.8256333157286304  0.6536856582091151
  0.5000000000000000  0.1743666842713695  0.3463143417908849
  0.7500000000000000  0.5000000000000000  0.7500000000000000
  0.2500000000000000  0.5000000000000000  0.7500000000000000
  0.5000000000000000  0.3256333157286305  0.8463143417908849
  0.5000000000000000  0.6743666842713696  0.1536856582091151
  0.5000000000000000  0.3256333157286305  0.1536856582091151
  0.5000000000000000  0.6743666842713696  0.8463143417908849
```

Table S3. Optimized VASP POSCAR for *Immm*-Re_3Zr.

Space group No. 71; 8 atoms per supplied cell; Direct fractional coordinates.

```
18:Immm(71)
   1.00000000000000
     3.1577429110717237    0.0000000000000000    0.0000000000000000
     0.0000000000000001    4.5499116313304384    0.0000000000000000
     0.0000000000000000    0.0000000000000000    9.0904193634140782
   Zr   Re
     2     6
Direct
  0.0000000000000000  0.5000000000000000 -0.0000000000000000
  0.5000000000000000  0.0000000000000000  0.5000000000000000
 -0.0000000000000000  0.0000000000000000  0.2436009280164271
```

```
  0.5000000000000000 -0.0000000000000000 -0.0000000000000000
  0.5000000000000000  0.5000000000000000  0.2563990719835728
  0.5000000000000000  0.5000000000000000  0.7436009280164271
  0.0000000000000000  0.5000000000000000  0.5000000000000000
 -0.0000000000000000  0.0000000000000000  0.7563990719835729
```

Table S4. Optimized VASP POSCAR for *P*4/*mmm*-Re_3Zr.

Space group No. 123; 4 atoms per supplied cell; Direct fractional coordinates.

```
1:P4/mmm(123)
   1.00000000000000
     3.1674800039527069   -0.0000000000000000   -0.0000000000000000
     0.0000000000000000    3.1674800039527073   -0.0000000000000000
    -0.0000000000000000    0.0000000000000000    6.5534496638234900
   Zr   Re
     1     3
Direct
  0.5000000000000000  0.5000000000000000  0.0000000000000000
  0.0000000000000000 -0.0000000000000000  0.2650845469746150
 -0.0000000000000000  0.0000000000000000  0.7349154530253849
  0.5000000000000000  0.5000000000000000  0.5000000000000000
```

Table S5. Optimized VASP POSCAR for *Pm*-3*n*-Re_3Zr.

Space group No. 223; 8 atoms per supplied cell; Direct fractional coordinates.

```
PY JOM2025
   1.00000000000000
     5.1125923651885596   -0.0000000000000000    0.0000000000000000
    -0.0000000000000000    5.1125923651885596   -0.0000000000000000
     0.0000000000000000    0.0000000000000000    5.1125923651885596
   Zr   Re
     2     6
Direct
 -0.0000000000000000  0.0000000000000000  0.0000000000000000
  0.5000000000000000  0.5000000000000000  0.5000000000000000
  0.5000000000000000  0.0000000000000000  0.2500000000000000
  0.5000000000000000  0.0000000000000000  0.7500000000000000
  0.2500000000000000  0.5000000000000000  0.0000000000000000
  0.7500000000000000  0.5000000000000000  0.0000000000000000
 -0.0000000000000000  0.2500000000000000  0.5000000000000000
 -0.0000000000000000  0.7500000000000000  0.5000000000000000
```

Table S6. Optimized VASP POSCAR for *Fm*-3*m*-Re_3Zr.

Space group No. 225; 16 atoms per supplied cell; Direct fractional coordinates.

```
2:Fm-3m(225)
   1.00000000000000
     6.3837057506535935   -0.0000000000000000   -0.0000000000000000
     0.0000000000000000    6.3837057506535935   -0.0000000000000000
     0.0000000000000000    0.0000000000000000    6.3837057506535935
   Zr   Re
     4    12
Direct
 -0.0000000000000000  0.0000000000000000  0.0000000000000000
 -0.0000000000000000  0.5000000000000000  0.5000000000000000
  0.5000000000000000  0.0000000000000000  0.5000000000000000
  0.5000000000000000  0.5000000000000000 -0.0000000000000000
  0.5000000000000000 -0.0000000000000000 -0.0000000000000000
  0.7500000000000000  0.7500000000000000  0.2500000000000000
  0.7500000000000000  0.7500000000000000  0.7500000000000000
  0.5000000000000000  0.5000000000000000  0.5000000000000000
  0.7500000000000000  0.2500000000000000  0.7500000000000000
  0.7500000000000000  0.2500000000000000  0.2500000000000000
 -0.0000000000000000  0.0000000000000000  0.5000000000000000
  0.2500000000000000  0.7500000000000000  0.7500000000000000
  0.2500000000000000  0.7500000000000000  0.2500000000000000
 -0.0000000000000000  0.5000000000000000 -0.0000000000000000
  0.2500000000000000  0.2500000000000000  0.2500000000000000
  0.2500000000000000  0.2500000000000000  0.7500000000000000
```
